# Mutant-selective topologic conversion facilitates selective degradation of a pathogenic prion isoform

**DOI:** 10.1038/s41418-019-0354-1

**Published:** 2019-05-24

**Authors:** Yumi Lee, Hongsik Eum, Duri Lee, Sohee Lee, Youngsup Song, Sang-Wook Kang

**Affiliations:** 1 Department of Biomedical Sciences, University of Ulsan College of Medicine, Seoul, Republic of Korea; 2 Asan Institute of Life Sciences, Asan Medical Center, Seoul, Republic of Korea

**Keywords:** Chaperones, Prions, Protein quality control

## Abstract

Regulating protein import across the endoplasmic reticulum (ER) membrane occasionally results in the synthesis of topologically unnatural variants, and their accumulation often leads to proteotoxicity. However, since this is a regulated process, it is questionable whether the topological rearrangement really has adverse consequences. In the present study, we provide an insight into the functional benefit of translocational regulation by illustrating mutant-selective topologic conversion (MSTC) and demonstrate that MSTC contributes to selective degradation of a membrane-anchored prion protein isoform (ctmPrP). We find that ctmPrP is inherently short-lived and topologically competent for degradation rather than accumulation. MSTC achieves, cotranslationally, the unique topology of ctmPrP during translocation, facilitating selective ctmPrP degradation from the ER via the proteasome-dependent pathway before entering the secretory pathway. At this time, the N-terminal polycationic cluster is essential for MSTC, and its cytosolic exposure acquires “ERAD-degron”-like activity for ctmPrP. Bypassing MSTC delays ctmPrP degradation, thus increasing prion proteotoxicity. Thus, topological rearrangement is used for the MSTC as a part of the protein quality control pathway to ensure the safety of the secretory pathway from misfolded PrP.

## Introduction

Approximately one-third of proteins, including secreted and transmembrane proteins, in mammalian cells are synthesized in the endoplasmic reticulum (ER). This synthesis is initiated by specific targeting of ribosome-bound nascent polypeptides to the ER membrane. On the ER membrane, nascent polypeptides are transferred to the Sec61 translocon protein channel and are then cotranslationally imported across the ER membrane (i.e., translocation). However, some proteins are often rejected from the translocon during translocation, resulting in the synthesis of topologically unnatural isoforms [1]. One such typical example is prion protein (PrP) [2, 3].

PrP is an enigmatic protein responsible for numerous neurodegenerative disorders (known as prion diseases) that affect both humans and animals with fatal outcomes. The conformational transition from the normal cellular form (PrP^C^) to a pathogenic, protease-resistant, and transmissible scrapie form (PrP^SC^) is believed to be the major risk-factor for all prion diseases [4]. However, neurodegenerative changes in prion diseases are not always correlated with accumulated PrP^SC^ in the brain [5], so PrP^SC^ may not be the only cause of neuronal dysfunction in prion diseases [6, 7]. This was suggested by early discoveries of topologically distinct PrP isoforms in various experimental systems and transgenic mice carrying certain disease-causing mutations [5, 8].

PrP is synthesized in three topologic isoforms at the ER membrane: secPrP, ntmPrP, and ctmPrP. While secPrP is the most abundant form that completely translocated into the ER membrane, ntmPrP and ctmPrP are minor species spanning the ER membrane once in opposite orientations, with the N-terminal region on the luminal and cytosolic side, respectively [3]. These topologic outcomes are achieved during translocation, in which the N-terminal signal sequence delivers the ribosome-associated PrP nascent chain to the ER (ER targeting) and opens the protein channel, Sec61 translocon, to pass the PrP (translocon gating) [9]. Of these two steps, translocon gating is the functional step that is coordinated by PrP mature domain and is influenced by the luminal environment. This step is often perturbed by ER stress, resulting in cotranslational rerouting of PrP nascent chain to the cytosol for degradation by the process known as *p*re-emptive *Q*uality *C*ontrol pathway (pQC) [10]. A similar mechanism is applied to the generation of ctmPrP whose unique topology is determined at the translocon gating [2].

ctmPrP is the only form that spontaneously causes neurodegeneration without PrP^SC^ accumulation [5]. This ctmPrP-induced neuronal dysfunction might be caused by the functional disruption of mahogunin (Mgrn1), an essential E3 ligase for neuronal viability, being inappropriately sequestrated to the cytosolically exposed N-terminal region of ctmPrP [11]. Thus, ctmPrP-induced neuronal dysfunction may be attributable to an aberrant topology rather than misfolding [12]. However, ctmPrP expression levels showed limited correlation with neurodegenerative phenotypes, in which ctmPrP was detected only in 20–30% of the total PrP in transgenic mice expressing a ctmPrP-favoring mutant [5, 13]. Comparable results were also seen in the inherited prion disease A117V (also known as Gerstmann–Sträussler–Scheinker disease) and verified in the brains of transgenic mice by post-mortem examinations [14]. These observations indicate there is an undefined cellular mechanism capable of selectively removing pathogenic ctmPrP.

Our comparative analyses of the topologic conversion, synthesis, turnover, localization, and biochemical properties of a ctmPrP-favoring mutant allow us to reveal the mutant-selective topologic conversion (MSTC) that is an underappreciated cellular strategy to selectively inhibit pathogenic ctmPrP expression. This study provides insight into how MSTC is regulated to influence the outcome of prion topology and proteotoxicity.

## Materials and methods

### Constructs, antibodies, and reagents

All mutant PrP constructs were generated from hamster *PRNP* cDNA (Genebank accession number: EF139168) that was cloned into the pcDNA5/FRT/TO vector (Invitrogen; Carlsbad, CA, USA) by site-directed mutagenesis using Phusion high-fidelity DNA polymerase (New England Biolabs; Ipswitch, MA, USA). Their mutations were verified by sequencing (Cosmogenetech; Seoul, South Korea). Fluorescent protein (FP) fusion constructs were created by inserting GFP or RFP genes into unique Bsu36I sites within the N-terminal-coding region of wild-type and mutant PrPs. A guide RNA construct of human Bag6 was engineered by inserting the target sequence (5′-GACCTTACTATCCCGGATGG-3′) into a unique BsmBI in the lentiGuide-Puro vector [15], a gift from Feng Zhang (Addgene; Watertown, MA, USA, plasmid # 52963). shRNA-targeting human p97 (TRCN0000339131) was purchased from Sigma-Aldrich (St. Louis, MO, USA). The following antibodies were used in this study: anti-Bag6 (Santa Cruz Biotechnology, Dallas, TX, USA, 1:1000 dilution), anti-p97 (Abcam; Cambridge, UK, 1:10,000), anti-PDI (PDI, 1:5000), and anti-BiP (BD; Franklin Lakes, NJ, USA, 1:1000). Anti-L7a (1:5000) and anti-Hsp90 (1:1000) antibodies were obtained from Cell Signaling Technology (Danvers, MA, USA). Anti-Sec61β (1:5000), TRAPα (1:2000), and anti-GFP (1:2000) antibodies have been previously described [16, 17]. We used two prion-specific antibodies with different epitopes: anti-PrP-A anti-serum (1:5000), which recognizes all mammalian species of PrP and SA-PrP [18]; and 3F4 antibody (BioLegend; San Diego, CA, USA, 1:10,000), which recognizes hamster and human PrP [19]. [^35^S]-methionine and trans-labeling mixture were purchased from PerkinElmer (Watham, MA, USA). Endo H, PNGase F, and all enzymes for cloning were from New England Biolabs. Trypsin, trypsin inhibitor, MG132, bafilomycin-A1, and all chemicals for biochemistry procedures were purchased from Sigma.

### In vitro analyses

DNA templates carrying the SP6 promoter sequence at their 5′ ends were PCR-amplified from PrP constructs and subjected to in vitro transcription with SP6 RNA polymerase. In vitro translation in rabbit reticulocyte lysate containing or lacking rough microsomes (RMs), followed by protease protection assay for topology determination, have been previously described [3, 20]. A series of ribosome-bound nascent PrP polypeptides were produced in the same manner from a defined length of truncated mRNA lacking a termination codon, and radioactive products were isolated via immunoprecipitation with 3F4 antibody to remove the interference of hemin on the gel.

### Cell culture analyses

**Fig. 1:**
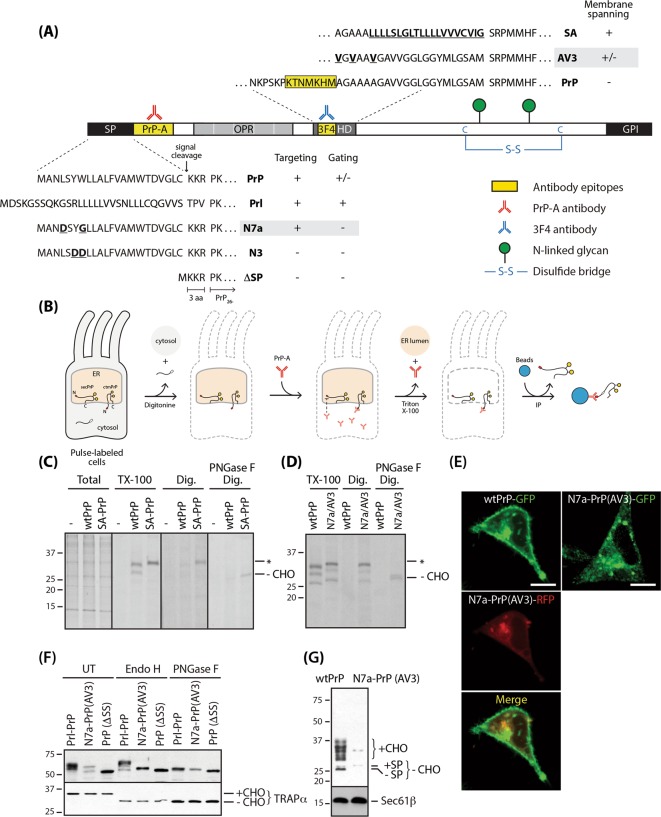
Analyses of the unique topology of ctmPrP. **a** Various mutations in the signal sequence (SP) and internal hydrophobic domain (HD) used in this study, the functional domain, and the antigenic determinants are illustrated. Differential ER targeting, gating, and membrane spanning efficiency distinguished mutants from wild type PrP [2, 3, 9]. N7a signal sequence and AV3 mutation in internal hydrophobic domain used in this study are in gray boxes. OPR octa-peptide repeats. C cysteine residue, FMD flanking mature domain. **b** Experimental strategy. Also see the section “Materials and methods”. **c** Cytosolically exposed N-terminal region of ctmPrP (marked with an asterisk*) was determined by immunoprecipitation with PrP-A anti-serum (3 µl/ml) in SP cells (expressing wtPrP and SA-PrP) with fully solubilized (TX-100) or intact ER membranes (Dig.), following pulse-labeling of the newly synthesized PrPs with [^35^S]-methionine for 15 min. The N-linked glycan modification of the C-terminal region was determined by monitoring whether the species captured by the antibody was sensitive to PNGase F (–CHO). Total total lysates extracted by TX-100. **d** wtPrP and N7a-PrP (AV3) (“N7a/AV3”) were analyzed as in **c**. **e** Subcellular localizations of PrPs were visualized in the HeLa cells transfected with wtPrP-GFP and N7a-PrP (AV3)-RFP, or N7a-PrP (AV3)-GFP alone. Scale bar: 10 µm. **f** Fully solubilized cells in **e** were diluted, digested by Endo H or PNGase F, and blotted with GFP antibody (1:2000). TRAPα (sensitive to both glycosidases) were used as a positive control. +CHO/−CHO: glycosylated/unglycosylated form. **g** Isogenic Flp-In T-Rex 293 stable cell lines expressing wtPrP and N7a-PrP (AV3) were generated. PrP expression was induced by doxycycline (100 ng/ml) for 12 h and confirmed by immunoblotting with 3F4 antibody (1:10,000). Equal loading was confirmed by blotting with anti-Sec61β antibody. +SP/−SP signal sequence uncleaved/cleaved forms

HeLa and Flp-In T-REx 293 cells were purchased from American Type Culture Collection (Manassas, VA, USA) and Invitrogen, respectively. Both cells were grown in Dulbecco’s Modified Eagle Medium (DMEM), supplemented with 10% fetal calf serum in 5% CO_2_ at 37 °C, and transfected with Lipofectamine 2000 (Invitrogen). Isogenic Flp-In T-REx 293 cell lines, expressing wild-type or mutant PrPs, were generated according to the manufacturer’s directions. In this system, the CMV promoter controlled PrP expression, induced by doxycycline (100 ng/ml) for 12 h unless otherwise indicated. Colony forming assays were performed using a previously published procedure with minor modifications [21]. Briefly, cells (100 cells per well) were plated on 35 mm dishes and cultured in the presence of doxycycline (100 ng/ml) for 3 weeks. Viable cell colonies were fixed, counter stained with 6% glutaraldehyde containing 0.5% crystal violet, and visualized via GelCount^TM^ (Oxford Optronix; Abington, UK) using the manufacturer’s image acquisition software. A Bag6-deficient cell line was produced using CRISPR/Cas9-mediated gene editing with Bag6-targeting sgRNA. As a negative control, we cloned additional Cas9 cells expressing non-targeting sgRNA. The Bag6 gene editing was verified using the T7E1-based heteroduplex cleavage assay, and its selective deficiency was confirmed by elimination of the Bag6 protein. Metabolic labeling of newly synthesized proteins, followed by immunoprecipitation with PrP-A anti-serum or 3F4 antibody, have been previously described [10, 22]. To determine the topology of PrP in vivo, pulse-labeled cells in 35 mm dishes were washed with 1x PBS and semi-permeabilized with 1 ml SP buffer (110 mM KOAc, 50 mM HEPES, 2 mM MgCl_2_, 0.015% digitonin) for 5 min on ice. The semi-permeabilized (SP) cells were further incubated with 1 ml SP buffer containing PrP-A anti-serum for 90 min at 4 °C. Following stringent washing with SP buffer to remove non-specifically bound or unbound antibody, SP cells were solubilized in 1 ml IP buffer (50 mM HEPES, 150 mM NaCl, 1% Triton X-100) and incubated with Protein G Mag Sepharose (GE Healthcare Life Sciences) for an additional 90 min at 4 °C. The beads were washed five times with 1 ml IP buffer and suspended in 20 µl 1.5x SDS–PAGE sample buffer (Fig. 1a). Subcellular localizations of PrPs were visualized in the cells transiently transfected with constructs expressing wild-type or mutant PrPs fused with FPs, as previously described [23], and images were obtained on a confocal microscope (Zeiss LSM780; Carl Zeiss Microimaging; Thornwood, NY, USA) using the manufacturer’s image acquisition software, ZEN 2012.

### Miscellaneous biochemistry

All experiments were performed in a cold chamber (4 °C) or on ice. For biochemical analyses of the solubilities and native sizes of PrP in detergent lysates, cells stably expressing wild type and mutant PrPs in 35 mm dishes were extracted with DB buffer (150 mM NaCl, 50 mM Tris, pH 7.4, 2 mM EDTA, 0.5% Triton X-100, 0.5% deoxycholate). After passing through a 22 G needle several times, the lysates were incubated for 30 min with rocking and centrifuged for 5 min at 10,000×*g* in a microcentrifuge. The supernatant was applied to a 2 ml linear 10–50% sucrose gradient prepared in DB buffer and centrifuged for 1 h at 55,000 rpm in a TLS-55 rotor (Beckman; Indianapolis, IN, USA). Two hundred microliters of fractions were collected from the top of the supernatant and analyzed via SDS–PAGE and immunoblotting with various antibodies.

RAMP isolation was performed according to previously published procedures with minor modifications [24, 25]. Briefly, cells cultured in 100 mm dishes and washed with 1x PBS were semi-permeabilized with 5 ml SP buffer and recovered via centrifugation for 5 min at 3000×*g*. SP cells were resuspended in 1 ml buffer S (20 mM Tris–HCl, pH 7.5, 5 mM MgCl_2_, 2 mM DTT, 300 mM NaCl, 2% digitonin) and incubated for 30 min at 4 °C with gentle rocking. Cell lysates were separated into supernatants (non-RAMPs) and pellets (RAMPs) via centrifugation for 1 h at 70,000 rpm in a TLA100.3 rotor (Beckman Coulter) using Optima MAX-XP Ultracentrifuge (Beckman Coulter), and the pellets were carefully resuspended in 1 ml buffer C (500 mM NaCl, 20 mM Tris–HCl, pH 7.4, 5 mM MgCl_2_, 1% TX-114) on ice. After incubation for 30 min at 4 °C with rocking, the samples were separated into supernatants and pellets via centrifugation for 1 h at 70,000 rpm. The pellets (ribosomes) were fully solubilized in 100 µl of buffer F (1% SDS, 100 mM Tris–HCl, pH 8.0). The supernatants (~1 ml) were incubated for 10 min at 37 °C and separated into the upper (soluble-RAMP) and lower phase (insoluble-RAMP) via centrifugation for 10 min at 13,500 rpm at RT. The upper phases (~800 µl) were carefully transferred to new tubes. The lower phases were washed twice in the same manner, followed by dilution in 1 ml of cold 0.5 M NaCl. Both the upper and lower phases were concentrated via TCA precipitation and fully solubilized in 100 µl of 1% SDS (in 100 mM Tris–HCl, pH 8.0) (Fig. S6). Comparative analyses of their biochemical properties, including glycosidase sensitivity, detergent solubility, trypsin sensitivity, and sucrose gradient centrifugation, were performed as previously described [10, 18, 19].

Exact times and conditions of each experiment are described in individual figure legends. Additional details of experimental methods can be found in the Supplemental Information.

## Results and discussion

### MSTC produces ctmPrP

To produce ctmPrP, we employed a well-characterized construct expressing a pathogenic PrP carrying a fatal mutation within its internal hydrophobic region (called PrP-AV3) (see Supplementary Note 5). We equipped it with an N-terminal signal sequence that is persistently controlled by the pre-emptive quality control (pQC) pathway (that is, N7a-PrP-AV3) [2, 26] (Fig. 1a). Intensive re-examination of this mutant in a cell-free translation system led us to propose the MSTC as a mechanism of ctmPrP generation (Fig. S1 and Supplementary Note 1).

MSTC also occurred in HeLa cells and produced ctmPrP in a similar manner in vitro. This was examined by detecting the unique topology of ctmPrP in semi-permeabilized cells after pulse-labeling (Fig. 1b and Supplementary Note 2). This experiment was validated with SA-PrP, a typical topologic ctmPrP marker [27] and revealed that three folding intermediates corresponding to non-glycosylated, mono-glycosylated, and di-glycosylated PrP synthesized from wtPrP and N7a-PrP-AV3 were recovered by PrP-A antibody when the ER was fully solubilized by TX-100 (~1%) (Fig. 1c). In contrast, when the ER remained intact, newly synthesized PrP was recovered selectively only in the cells expressing N7a-PrP-AV3 by the antibody and characterized as ctmPrP with its N-linked glycan moieties fully digested by PNGase F (Fig. 1d).

In addition to its unique topology, ctmPrP appeared to have several important features distinguishing it from secPrP as follows. First, wtPrP was displayed primarily on the cell surface and minimally in the intracellular compartments, whereas N7a-PrP-AV3 was not displayed on the cell surface but was weakly visualized throughout the ER of a limited number of cells (Fig. 1e). Second, ctmPrP was glycosylated, but not fully modified by Golgi enzymes, as shown by its sensitivity to digestion by endoglycosidase H (Endo H) that selectively cleaves simple modifications of N-linked glycans processed by ER enzymes (Fig. 1f). Third, the level of newly synthesized ctmPrP was similar to that of wtPrP (Fig. 1d), but extremely low protein levels had accumulated in the cells (Fig. 1g). This discrepancy in between the levels of ctmPrP newly synthesized and accumulated in the cells seems to be caused by selective degradation in a proteasome-dependent manner at the post-translational level, as shown by the recovery of ctmPrP levels by MG132 (Fig. S3A) rather than by BAF-A1 (Fig. S3B). At last, the N-terminal signal sequence and C-terminal GPI-anchored sequence remained uncleaved (Fig. S4A–S4E). This is the reason for the slower migration of N7a-PrP-AV3 on the gel than that of wtPrP (Fig. 1d). The unprocessed signal sequence and GPI-anchored sequence were discovered later as important requirements for the synthesis of ctmPrP (Fig. S4). All these unique features suggest altered metabolism and trafficking of ctmPrP in the early secretory pathway.

### ctmPrP is an ERAD substrate

**Fig. 2:**
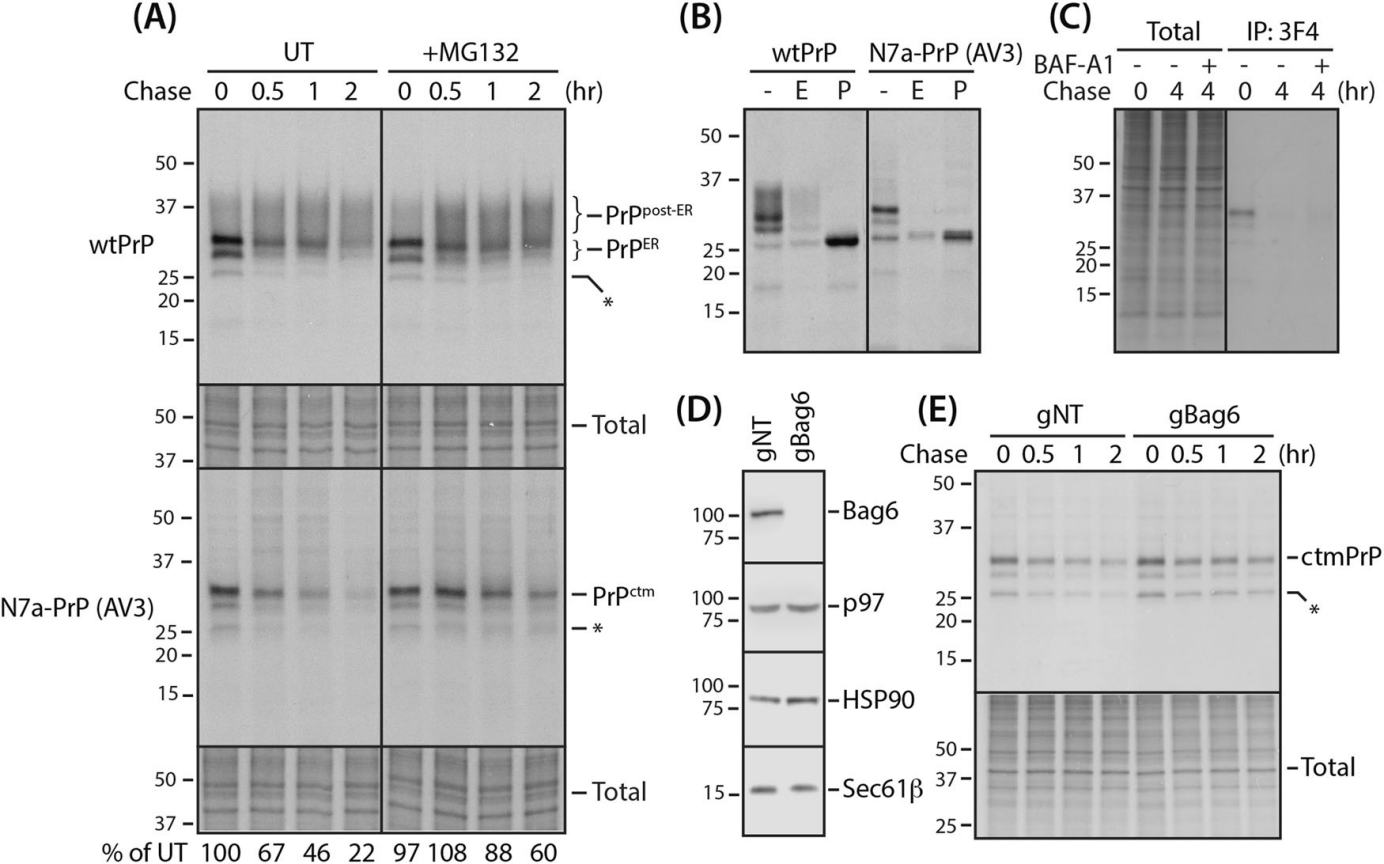
Analyses of ctmPrP synthesis and degradation. **a** Stable cell lines, expressing wtPrP and N7a-PrP (AV3) (Fig. 1f), were pulse-labeled for 30 min and harvested at the indicated time points during the chase in the presence and absence of MG132 (5 µM). The cells were fully solubilized and subjected to immunoprecipitation with 3F4 antibody (1:1000 dilution). The ctmPrP density was quantified by Image J software (NIH) and expressed as a percentage of the amount of PrP labeled at pulse. *Unglycosylated form, PrP^ER^: ER form, PrP^post-ER^: fully glycosylated post-ER form, Total: input. **b** Pulse-labeled PrPs captured by 3F4 antibody in **a** were digested by Endo H (E) or PNGase F (P) for 4 h. **c** Pulse-labeled cells expressing N7a-PrP-AV3 were harvested at time 0 and 4 h during the chase in the presence and absence of BAF-A1 (100 ng/ml). Immunoprecipitation was performed as in **a**. **d** Bag6-targeting CRISPR/Cas9 cells expressing N7a-PrP (AV3) were generated. Selective Bag6 depletion was confirmed by immunoblotting with anti-Bag6 antibody (1:1000). gNT non-target gRNA used as a negative control. **e** ctmPrP turnover rates in Bag6-deficient CRISPR/Cas9 cells were assessed as in **a**

PrP species with features similar to ctmPrP have been discovered in GPI-anchor-deficient cells, in which PrP-bearing uncleaved GPI-anchored sequences failed to exit the ER, and were instead degraded by ER-associated protein degradation (ERAD) [18]. Given that ctmPrP also has an uncleaved GPI-anchored sequence (Fig. S4D), we hypothesized that ctmPrP may be metabolized in a similar manner. This hypothesis was verified by the spatiotemporal analyses of newly synthesized ctmPrP (Fig. 2).

Ordinarily, secPrP is core-glycosylated, folded in the ER lumen, and further glycosylated in the Golgi. The properly folded PrP is sorted to the cell surface, endocytosed, and eventually degraded in the lysosome [23]. In a typical pulse-chase experiment (Fig. 2a), wtPrP was shown to be synthesized, modified, and trafficked in this normal PrP metabolism. However, ctmPrP spontaneously and rapidly degraded with a *t*_1/2_ of ~1 h without an appearance of the fully matured glycosylated form that is resistant to Endo H (Fig. 2b). Consistent with the immunoblotting result (Fig. S3A and B), ctmPrP was sensitive to proteasome, but not lysosome (Fig. 2c), as shown by its delayed selective degradation (*t*_1/2_ of >2 h) by MG132 (Fig. 2a). By contrast, secPrP was resistant to the proteasome inhibitor because its general metabolism and trafficking was unchanged (Fig. 2a). Delayed degradation of ctmPrP was also seen upon proteasome disassembly induced by the depletion of Bag6 [28] (Fig. 2d and Supplementary Note 3) and verified by pulse-chase experiments in a similar manner (Fig. 2e). Taken together, these data suggest that ctmPrP is an ERAD substrate that is spontaneously degraded by proteasome-dependent pathway.

### N-terminal polycationic cluster of ctmPrP acts as an ER-retention signal and an ERAD-degron

**Fig. 3:**
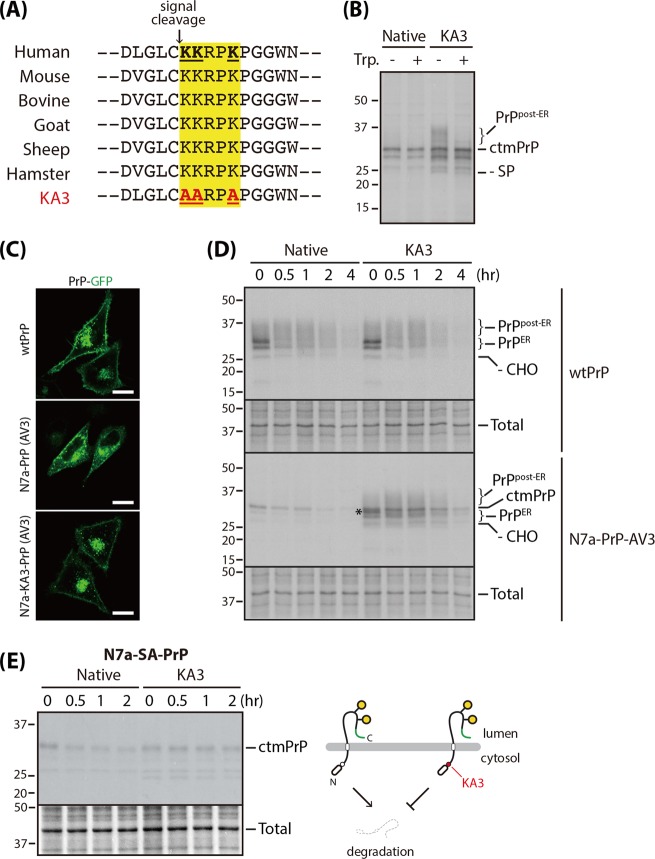
Effect of N-terminal polycationic cluster on ctmPrP metabolism and trafficking. **a** Comparison of the N-terminal amino acid sequence around the signal sequence cleavage site of PrP in vertebrates. The polycationic cluster is boxed in yellow. The KA3 mutant was generated by replacing three lysine residues with alanines. **b** Cells expressing native ctmPrP and the KA3 mutant were pulse-labeled in DMEM supplemented with 10% dialyzed FBS, 0.6 µM methionine, and 2 µM cysteine for 12 h. Cells were incubated for 1 h, with or without trypsin (0.25%) on ice. The trypsin was inactivated by the trypsin inhibitor (250 µg/ml). The cells were fully solubilized and subjected to immunoprecipitation with 3F4 antibody as in Fig. 2a. **c** Subcellular localizations of PrP in the cells were analyzed in HeLa cells transfected with the indicated constructs as in Fig. 1d. Scale bar: 10 µm. **d** PrP synthesis, degradation, and processing were analyzed in cells stably expressing the indicated wild-type and mutant PrPs. Pulse-chase experiments, followed by immunoprecipitation with anti-3F4 antibody, were performed as in Fig. 2a. *Glycosylated secPrP in the ER (both signal sequence and GPI-anchored sequence appeared to be processed properly). **e** Effect of KA3 mutation on ctmPrP synthesis and degradation in the cells expressing N7a-SA-PrP carrying wild type (native) or mutant (KA3) polycationic cluster was assessed by pulse-chase experiment followed by immunoprecipitation with PrP-A antibody as in Fig. 2a (left panel). Inhibitory effect of cytosolically exposed KA3 mutation on ctmPrP degradation is illustrated (right panel)

Our finding that ctmPrP is retained in the ER raised the possibility of the existence of a sequence motif responsible for ER retention. Unlike other PrP isoforms, the N-terminal region of ctmPrP is exposed to cytosol, so we looked for such a signal motif within this region. In this investigation, we noticed that a short di-lysine (-KKXX-) motif (often referred to as polycationic cluster), a well-known ER-retention signal for several transmembrane proteins [29], within the flanking mature domain of PrP could be exposed to cytosol by MSTC. To examine whether this motif indeed acts as an ER-retention signal, we engineered a construct expressing the KA3 mutant, in which three highly conserved lysine residues in the polycationic cluster (-**KK**RP**K**-) were replaced with alanines (-**AA**RP**A**-) (Fig. 3a). This mutation was introduced into the wtPrP and N7a-PrP-AV3 constructs, and their expressions were analyzed in isogenic Flp-In 293T-Rex stable cell lines.

Of the two PrP constructs, only N7a-PrP-AV3 was sensitive to the KA3 mutation, resulting in the synthesis of fully glycosylated form (when cells were pulse-labeled for 12 h) that was selectively digested by exogenously added trypsin (Fig. 3b). Apparently, GFP-fused KA3 mutant was redistributed from the ER to the Golgi and the cell surface [30] (Fig. 3c). Topologic conversion of ctmPrP to secPrP mediated by the disruption of di-lysine motif has been shown to be the reason for this observation (Fig. S5B). This observation hinted at the possibility that cytosolically exposed polycationic cluster plays a role as an ER-retention signal for the ctmPrP.

During the course of this study, we noticed that ctmPrP was also increased unexpectedly by KA3 mutation (Fig. 3d, b). This paradoxical result provides the possibility that KA3 mutation perturbs additional steps in ctmPrP degradation. Comparative analyses of the synthesis and turnover rates of the native PrP vs. the KA3 mutant revealed that the KA3 mutation did not affect endocytic clearance of secPrP [31] or ctmPrP. This was demonstrated by the similar turnover rate of their fully glycosylated species upon chase. In the analysis of newly synthesized folding intermediates of ctmPrP bearing the KA3 mutation, we found an additional product displaying similar gel mobility to the ER form of secPrP at an early time point. This species seemed to be a GPI-anchored folding intermediate lacking the ER signal sequence, as it was rapidly metabolized into the fully glycosylated form at a similar rate to the ER form of secPrP. Despite the KA3 mutation, ctmPrP was still synthesized, but not degraded as rapidly as native ctmPrP, and remained until 2 h after chase (Fig. 3d). Similar results were also shown by the disruption of the polycationic cluster in a typical ctmPrP marker, SA-PrP, the turnover rate of which is very similar to that of N7a-PrP-AV3; however, its translocation is not increased by KA3 mutation (Fig. 3e). Therefore, we reasoned that the delayed degradation of ctmPrP is caused by the disrupted cytosolic polycationic cluster acting as an amino-terminal degradation signal [32, 33].

Taken together, our results suggest that the cytosolically exposed N-terminal polycationic cluster functions both as an ER-retention signal and a degradation signal in a manner similar to that of the heavy chain of secretory immunoglobulin M (sIgM) [34]. This combined regulation ensures pathogenic ctmPrP does not accumulate in the secretory pathway.

### MSTC requires N-terminal polycationic cluster

**Fig. 4:**
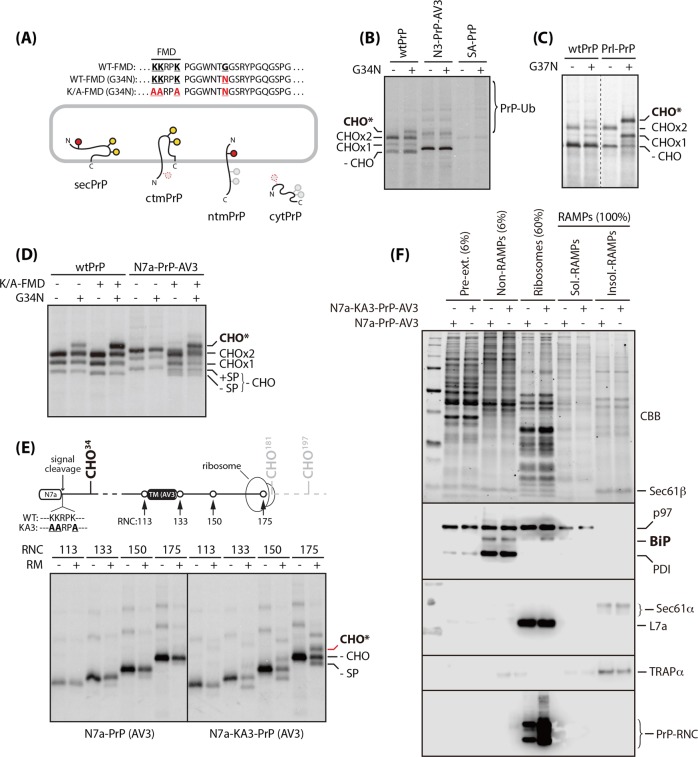
Effect of N-terminal polycationic cluster on MSTC. **a** Compartment-restricted glycosylation assay. Expected N-glycan modifications were illustrated with different colors. Red circles: G34N N-glycan acceptor site, black circles filled with red: glycosylated G34N, black circles filled with yellow: native N-glycan acceptor site. **b**, **c**, and **d** The assay system was validated by the modification of all three glycan acceptor sites, including G34N (“CHO*”), of wild-type PrP expressed in pulse-labeled cells. Immunoprecipitation of PrP expressed in pulse-labeled cells expressing the indicated wild-type and mutant PrPs carrying cytosolic N-terminal region were performed as described in Fig. 2a. **e** Lengths of truncated mRNA lacking termination codon (upper panel) were transcribed, translated, and immunoprecipitated (lower panel), in vitro, as described in the section “Materials and methods”. **f** Cells stably expressing the indicated mutant PrPs were extracted and fractionated as described in the section “Materials and methods” and analyzed on the gel. The fractionation fidelity was determined by immunoblotting with antibodies of typical marker proteins

To determine whether the MSTC may be regulated by the polycationic cluster, we employed a compartment-restricted glycosylation assay that could readily detect whether the N-terminal site can access the ER lumen (Fig. 4a). This assay was validated by monitoring the selective modification of an N-linked glycosylation acceptor site (G34N, hereafter referred to as CHO*) that was introduced into the N-terminal region of wtPrP (Fig. 4b) and verifying increased CHO* attributable to enhanced translocation efficiency (by replacing the native signal sequence of PrP with that of prolactin) (Fig. 4c). As negative controls, we used N3-PrP-AV3; N3 signal is defective in ER targeting, which results in PrP being largely in the cytosol [2, 9] (see Fig. S1A and B) and SA-PrP whose G34N residues could not access luminal side (Fig. 4b).

Once validated, we subjected the KA3 mutants to this approach. As shown by an appearance of CHO* (Fig. 4d), the cytosolic N-terminal region of N7a-PrP-AV3 appeared to be flipped to the luminal side by KA3 mutation, suggesting the interference of MSTC. However, none of the three lysine residues was shown not to be functionally more important, instead the number of lysine residues was positively correlated with CHO* levels (Fig. S5A and S5B). The lysine mutation appears to cause a certain degree of structural change owing to steric constraints, as previously suggested [31]. Thus, MSTC requires proper assembly of lysine residues in the N-terminal polycationic cluster.

To better understand the KA3 mutation effect on the topologic conversion of N7a-PrP-AV3 from ctmPrP to secPrP during translocation, we turned to in vitro analysis. To determine CHO* at distinct translocation stages, we synthesized a series of defined radiolabeled nascent chains that associated with ribosomes. These were readily translated from truncated mRNA, lacking the termination codon in rabbit reticulocyte lysate (Fig. 4e). When analyzed in the presence of rough microsomes (RMs) derived from HeLa cells, CHO* was selectively observed in translocation intermediates (of 133 and 175 residues) of KA3 mutants, and progressively increased as synthesis progressed. Additional evidence to demonstrate the luminal N-terminus of KA3 mutant was provided by the synthesis of translocation intermediates lacking signal sequences.

To investigate whether this effect also occurs in vivo, we analyzed ribosome-bound nascent chains (RNCs) isolated from cells expressing N7a-PrP-AV3 containing a native or mutant polycationic cluster (Fig. S6 and Supplementary Note 4). Our observation that a similar amount of translocon core components, derived from native PrP and the KA3 mutant, were recovered, suggests that efficient ribosome delivery onto the ER membrane is not affected by the polycationic cluster disruption. This was further supported by the similar level of the ribosomal protein, L7a. Nevertheless, an increased level of RNC was consistently observed in the KA3 mutant (Fig. 4f). Together with a number of in vitro studies demonstrating that PrP translocation and topogenesis are controlled at the gating step rather than the targeting step [2, 9], post-targeting regulation of MSTC appears to be the reason for this discrepancy.

BiP and PDI have been shown to bind directly to nascent proteins entering the ER and assist their cotranslational folding [10, 35, 36]. Therefore, we reasoned that, by monitoring the interaction of these proteins with nascent proteins, we could gain insight into the effect of KA3 mutation on MSTC in vivo. Comparative analysis of the distribution of BiP and PDI in our fractionations revealed both proteins were found primarily in the ribosome-unbound membrane fraction (“Non-RAMP”). However, a substantial amount of BiP (but not PDI) was also found selectively in the ribosome fraction enriched in the RNC of the KA3 mutant, suggesting the interaction of BiP and the KA3 mutant during translocation. The spatial perturbation of this interaction seems to be the potential MSTC mechanism. Since reduced, functionally available BiP in the ER lumen is the central mechanism of substrate-specific attenuation of protein translocation into the ER during stress, MSTC seems to be a physiologically relevant process as part of the pQC pathway [10].

### Bypassing MSTC for ctmPrP elicits proteotoxic effects

In the present study, we found a pathway, defined as MSTC, which induced topologic conversion of proteotoxic PrP-AV3 (see Supplementary Note 5) from secPrP to ctmPrP. Since ctmPrP is an ERAD substrate that is rapidly degraded in a proteasome-sensitive manner (Fig. 2), we hypothesized that MSTC may be a protective pathway against misfolded PrP. To investigate this hypothesis, we used PrP-AV3 as a pathogenic reporter and investigated the consequence of MSTC for PrP-AV3. To this end, we generated cell lines stably expressing either N7a-PrP-AV3 or the KA3 mutant that were constitutively regulated or not by MSTC, respectively (hereafter, referred to as “MSTC” or “non-MSTC” cells, respectively) (also see Fig. 3).

**Fig. 5:**
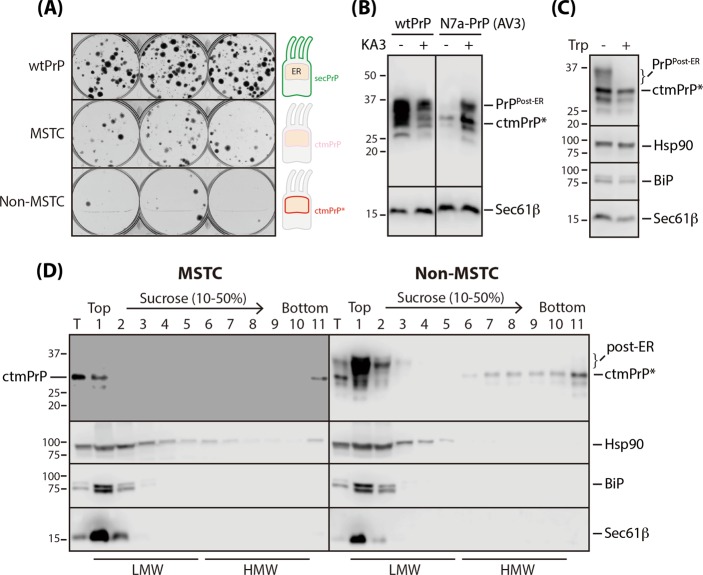
Bypassing effect of MSTC on PrP proteotoxicity. **a** Cells (100 cells/well) stably expressing wtPrP, N7a-PrP-AV3 (MSTC), and the KA3 mutant (non-MSTC) were plated in triplicate and visualized 3 weeks later by staining with crystal violet. **b** Cell stably expressing wild-type and mutant PrPs as indicated were fully solubilized with 1% SDS, and PrP expression was assessed via immunoblotting with 3F4 antibody. Equal loading was determined by measuring the Sec61β expression level. ctmPrP*: ctmPrP form synthesized by the KA3 mutant. **c** Selective digestion of PrP on the surface of the cells expressing the KA3 mutant was performed as in Fig. 3b and monitored via immunoblotting as in **b**. **d** The differential solubility and native size of the ctmPrP forms detected in MSTC and non-MSTC cells were determined using a sucrose gradient as described in the section “Materials and methods”. LMW low-molecular-weight fractions, HMW high-molecular-weight fractions

Notably, non-MSTC cells were less viable than MSTC cells (Fig. 5a). This was demonstrated using colony forming assays in which genes encoding MSTC and non-MSTC substrates were homogeneously transcribed by doxycycline. Consistent with our pulse-labeling experimental results (Fig. 3b), PrP-AV3 was accumulated as the fully glycosylated form with increased ctmPrP-like subpopulation in non-MSTC cells (similar in size to the ctmPrP population detected in MSTC cells). Conversely, it was mostly (but not entirely) degraded in MSTC cells (Fig. 5b). Fully glycosylated species were completely digested by exogenously added trypsin, indicating its cell surface expression. In contrast, the ctmPrP-like subpopulation was mostly resistant to trypsin digestion because of its retention in intracellular compartments (Fig. 5c). Further support for this observation was provided by the pulse-chase experiment, indicating that a substantial amount of this subpopulation, newly synthesized from the KA3 mutant, still remains without being fully glycosylated up to 4 h after chase (Fig. 3d). Therefore, we considered this ctmPrP-like subpopulation, hereafter referred to as ctmPrP*, as the cytotoxic species in non-MSTC cells.

Importantly, the ctmPrP* that was metabolized differently than native ctmPrP was indeed folded differently, as indicated by comparative analyses of their solubilities and native sizes in detergent lysates using a sucrose gradient (Fig. 5d). Although the native ctmPrP expression level was consistently very low, about half was recovered in the “soluble” and half in the “insoluble” fractions (the top and bottom fractions of the sucrose gradient, respectively). In contrast, ctmPrP* has been shown to be heterogeneously oligomerized and was recovered from bottom fractions (fractions 6–1 of the sucrose gradient), the high-molecular-weight fractions of the sucrose gradient. To investigate whether the chaperones’ biochemical state changes depending on ctmPrP’s folding state [10], we monitored BiP and Hsp90 recovery in the sucrose gradient. In both cell lines, BiP, a general chaperone in the ER, was recovered primarily in the same soluble fractions of the sucrose gradient (shown not to interact with ctmPrP*, because BiP and ctmPrP* were recovered in different fractions). However, Hsp90, a cytosolic chaperone, was recovered with ctmPrP (but not with ctmPrP*) in the bottom fraction collected from MSTC cells. Given the fact that Hsp90 is required for the degradation of several membrane proteins in the ER [37, 38], this observation hints at the possibility that selective proteasomal delivery of ctmPrP for degradation could be mediated by the role of Hsp90 interacting with the polycationic cluster within cytosolically exposed N-terminal region of ctmPrP. By contrast, the failure of the interaction by the disruption of polycationic cluster leads to the accumulation of ctmPrP in the ER. ctmPrP is prone to aggregate when it fails to be degraded because it carries a cytosolically exposed uncleaved hydrophobic signal sequence. The ctmPrP aggregate promotes inappropriate sequestration and functional depletion of essential proteostasis regulators such as Mgrn [11] (also see Supplementary Note 6). This may be a worthwhile scenario for prion pathogenesis caused by the failure of MSTC. Overall, since impaired metabolism, incomplete glycan modification, cell surface inaccessibility, and detergent insolubility are all indirect indicators of PrP misfolding [19], ctmPrP* appears to be misfolded, and its accumulation without degradation gives rise to prion proteotoxicity.

**Fig. 6:**
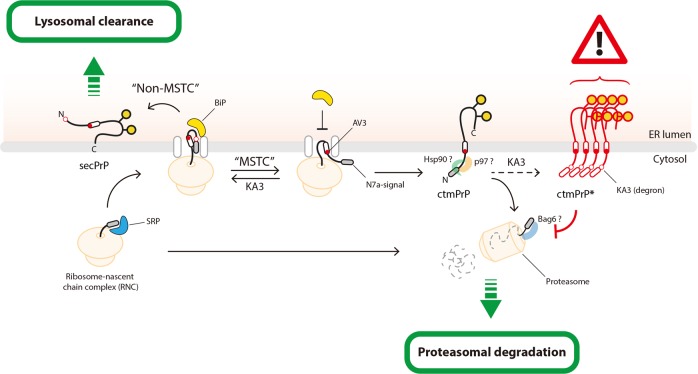
Working model depicting the underlying mechanism of MSTC-mediated prion homeostasis. PrP is a GPI-anchored protein that is poorly degraded by the proteasome-dependent pathway even if it is misfolded. In the present study, we propose that ctmPrP, in contrast to other PrP isoforms, is an ERAD substrate that is spontaneously extracted from the ER and degraded via the proteasome-dependent pathway. Several pathogenic PrP mutants whose mutations occur within the internal hydrophobic region (that is, the TMD; AV3 in this study) spontaneously undergo MSTC. Mechanistically, MSTC spatially interferes with the interaction of nascent PrP polypeptide with BiP, resulting in topologic conversion of their N-terminal region to the cytosolic side. The polycationic cluster within the N-terminal region exposed to the cytosol not only plays an important role as a topologic determinant during this process, but also acts as an N-terminal degradation signal for ctmPrP when exposed to the cytosol. The cytosolically exposed polycationic cluster is shown to recruit degradation machinery, Hsp90, p97, Bag6, and other unidentified cytosolic factors. However, these trans-acting factors can be inappropriately recruited and thus functionally depleted by the accumulation of ctmPrP. This is a potential mechanism for the pathogenic consequence induced by ctmPrP accumulation

In conclusion, we illustrate here that ctmPrP is an intrinsically degradable species rather than proteotoxic and is generated by MSTC. The defects induce the ctmPrP accumulation responsible for prion proteotoxicity (Fig. 6). In the present study, we discovered a topologic sequence (polycationic cluster) required for MSTC within the cytosolically exposed N-terminal region of ctmPrP (see Supplementary Note 7). Cytosolic polycationic cluster is provided both as an ER-retention signal and as an amino-terminal degradation signal, like a degron, for ctmPrP. Eventually, ctmPrP is discriminated from other PrP isoforms and rapidly degraded via the proteasome-dependent pathway (see Supplementary Note 8). Disruption of this motif (as in KA3) inhibits MSTC, allowing ctmPrP to be liberated from the ER, metabolized through the secretory pathway, and secreted on the cell surface (Fig. 3). Nevertheless, residual ctmPrP (ctmPrP*) can be generated, but is not extracted as efficiently as native ctmPrP from the ER because it lacks the N-terminal polycationic cluster (Figs. 3 and 5). Collectively, the cytosolically exposed N-terminal polycationic cluster is functionally involved throughout the series of events that generate, discriminate, and eliminate pathogenic ctmPrP, ensuring the safety of the secretory pathway from misfolded PrP (see Supplementary Note 9).

To better understand the molecular mechanism by which ctmPrP is triaged from the ER, further studies must still be conducted to identify cofactors specifically interacting with the cytosolically exposed polycationic cluster. Our primary interest is Hsp90, whose functional depletion, resulting from inappropriate sequestration to the N-terminal polycationic cluster, may interfere with the assembly of oligomeric complexes that target client proteins for degradation (through mechanisms such as p97 and the 26S proteasome), and eventually disrupt the overall proteostasis network [39–41]. We are also interested in cooperative regulation of luminal (BiP) and cytosolic chaperones (Hsp90) for ctmPrP degradation, as shown for CFTR [42]. Since ctmPrP is extremely labile, comparative analysis of the enriched fractions of ribosome-bound-nascent ctmPrP polypeptides isolated from the MSTC and non-MSTC cells (Fig. 4f) will facilitate these future studies.

## Supplementary information

Supplementary Notes

Supplementary Figure Legends

Supplementary Figure 1

Supplementary Figure 2

Supplementary Figure 3

Supplementary Figure 4

Supplementary Figure 5

Supplementary Figure 6

Supplementary Figure 7

## References

[1] Hegde RS, Kang SW (2008). The concept of translocational regulation. J cell Biol.

[2] Kim SJ, Rahbar R, Hegde RS (2001). Combinatorial control of prion protein biogenesis by the signal sequence and transmembrane domain. J Biol Chem.

[3] Kim SJ, Hegde RS (2002). Cotranslational partitioning of nascent prion protein into multiple populations at the translocation channel. Mol Biol cell.

[4] Prusiner SB (1998). Prions. Proc Natl Acad Sci USA.

[5] Hegde RS, Mastrianni JA, Scott MR, DeFea KA, Tremblay P, Torchia M (1998). A transmembrane form of the prion protein in neurodegenerative disease. Science.

[6] Prusiner SB (1997). Prion diseases and the BSE crisis. Science.

[7] Weissmann C (1995). Molecular biology of transmissible spongiform encephalopathies. Prog Brain Res.

[8] Hay B, Barry RA, Lieberburg I, Prusiner SB, Lingappa VR (1987). Biogenesis and transmembrane orientation of the cellular isoform of the scrapie prion protein [published errratum appears in Mol Cell Biol 1987 May;7(5):2035]. Mol Cell Biol.

[9] Kim SJ, Mitra D, Salerno JR, Hegde RS (2002). Signal sequences control gating of the protein translocation channel in a substrate-specific manner. Dev Cell.

[10] Kang SW, Rane NS, Kim SJ, Garrison JL, Taunton J, Hegde RS (2006). Substrate-specific translocational attenuation during ER stress defines a pre-emptive quality control pathway. Cell.

[11] Chakrabarti O, Hegde RS (2009). Functional depletion of mahogunin by cytosolically exposed prion protein contributes to neurodegeneration. Cell.

[12] Prusiner SB, Scott MR (1997). Genetics of prions. Annu Rev Genet.

[13] Rane NS, Chakrabarti O, Feigenbaum L, Hegde RS (2010). Signal sequence insufficiency contributes to neurodegeneration caused by transmembrane prion protein. J Cell Biol.

[14] Asante EA, Linehan JM, Smidak M, Tomlinson A, Grimshaw A, Jeelani A (2013). Inherited prion disease A117V is not simply a proteinopathy but produces prions transmissible to transgenic mice expressing homologous prion protein. PLoS Pathog.

[15] Sanjana NE, Shalem O, Zhang F (2014). Improved vectors and genome-wide libraries for CRISPR screening. Nat Methods.

[16] Snapp EL, Reinhart GA, Bogert BA, Lippincott-Schwartz J, Hegde RS (2004). The organization of engaged and quiescent translocons in the endoplasmic reticulum of mammalian cells. J Cell Biol.

[17] Fons RD, Bogert BA, Hegde RS (2003). Substrate-specific function of the translocon-associated protein complex during translocation across the ER membrane. J Cell Biol.

[18] Ashok A, Hegde RS (2008). Retrotranslocation of prion proteins from the endoplasmic reticulum by preventing GPI signal transamidation. Mol Biol Cell.

[19] Ashok A, Hegde RS (2009). Selective processing and metabolism of disease-causing mutant prion proteins. PLoS Pathog.

[20] Sharma A, Mariappan M, Appathurai S, Hegde RS (2010). In vitro dissection of protein translocation into the mammalian endoplasmic reticulum. Methods Mol Biol.

[21] Franken NA, Rodermond HM, Stap J, Haveman J, van Bree C (2006). Clonogenic assay of cells in vitro. Nat Protoc.

[22] Choi I, Kim J, Park JY, Kang SW (2013). Cotransin induces accumulation of a cytotoxic clusterin variant that cotranslationally rerouted to the cytosol. Exp Cell Res.

[23] Satpute-Krishnan P, Ajinkya M, Bhat S, Itakura E, Hegde RS, Lippincott-Schwartz J (2014). ER stress-induced clearance of misfolded GPI-anchored proteins via the secretory pathway. Cell.

[24] Shibatani T, David LL, McCormack AL, Frueh K, Skach WR (2005). Proteomic analysis of mammalian oligosaccharyltransferase reveals multiple subcomplexes that contain Sec61, TRAP, and two potential new subunits. Biochemistry.

[25] Conti BJ, Devaraneni PK, Yang Z, David LL, Skach WR (2015). Cotranslational stabilization of Sec62/63 within the ER Sec61 translocon is controlled by distinct substrate-driven translocation events. Mol Cell.

[26] Rane NS, Kang SW, Chakrabarti O, Feigenbaum L, Hegde RS (2008). Reduced translocation of nascent prion protein during ER stress contributes to neurodegeneration. Dev Cell.

[27] Emerman AB, Zhang ZR, Chakrabarti O, Hegde RS (2010). Compartment-restricted biotinylation reveals novel features of prion protein metabolism in vivo. Mol Biol Cell.

[28] Akahane T, Sahara K, Yashiroda H, Tanaka K, Murata S (2013). Involvement of Bag6 and the TRC pathway in proteasome assembly. Nat Commun.

[29] Vincent MJ, Martin AS, Compans RW (1998). Function of the KKXX motif in endoplasmic reticulum retrieval of a transmembrane protein depends on the length and structure of the cytoplasmic domain. J Biol Chem.

[30] Stewart RS, Harris DA (2005). A transmembrane form of the prion protein is localized in the Golgi apparatus of neurons. J Biol Chem.

[31] Khalife M, Reine F, Paquet-Fifield S, Castille J, Herzog L, Vilotte M (2016). Mutated but not deleted ovine PrP(C) N-terminal polybasic region strongly interferes with prion propagation in transgenic mice. J Virol.

[32] Bachmair A, Finley D, Varshavsky A (1986). In vivo half-life of a protein is a function of its amino-terminal residue. Science.

[33] Ravid T, Hochstrasser M (2008). Diversity of degradation signals in the ubiquitin-proteasome system. Nat Rev Mol Cell Biol.

[34] Shapira I, Charuvi D, Elkabetz Y, Hirschberg K, Bar-Nun S (2007). Distinguishing between retention signals and degrons acting in ERAD. J Cell Sci.

[35] Cesaratto F, Sasset L, Myers MP, Re A, Petris G, Burrone OR (2018). BiP/GRP78 mediates ERAD targeting of proteins produced by membrane-bound ribosomes stalled at the STOP-codon. J Mol Biol.

[36] Matlack KE, Misselwitz B, Plath K, Rapoport TA (1999). BiP acts as a molecular ratchet during posttranslational transport of prepro-alpha factor across the ER membrane. Cell.

[37] Fuller W, Cuthbert AW (2000). Post-translational disruption of the delta F508 cystic fibrosis transmembrane conductance regulator (CFTR)-molecular chaperone complex with geldanamycin stabilizes delta F508 CFTR in the rabbit reticulocyte lysate. J Biol Chem.

[38] Theodoraki MA, Caplan AJ (2012). Quality control and fate determination of Hsp90 client proteins. Biochim Biophys Acta.

[39] Yang C, Wang H, Zhu D, Hong CS, Dmitriev P, Zhang C (2015). Mutant glucocerebrosidase in Gaucher disease recruits Hsp27 to the Hsp90 chaperone complex for proteasomal degradation. Proc Natl Acad Sci USA.

[40] Young JC, Agashe VR, Siegers K, Hartl FU (2004). Pathways of chaperone-mediated protein folding in the cytosol. Nat Rev Mol Cell Biol.

[41] McClellan AJ, Xia Y, Deutschbauer AM, Davis RW, Gerstein M, Frydman J (2007). Diverse cellular functions of the Hsp90 molecular chaperone uncovered using systems approaches. Cell.

[42] Wang X, Venable J, LaPointe P, Hutt DM, Koulov AV, Coppinger J (2006). Hsp90 cochaperone Aha1 downregulation rescues misfolding of CFTR in cystic fibrosis. Cell.

