# Supplementary material for: Mutant-selective topologic conversion facilitates selective degradation of a pathogenic prion isoform
Source: Cell Death Differ. 2019 May 24;27(1):284–96. doi: 10.1038/s41418-019-0354-1 (PMC7205900; doi:10.1038/s41418-019-0354-1)
Supplement: Supplementary file 1 — Supplementary Notes [file 41418_2019_354_MOESM1_ESM.docx]

**[Supplemental Notes]**

**Mutant-selective topologic conversion facilitates selective degradation of a pathogenic prion isoform**

Yumi Lee, Hongsik Eum, Duri Lee, Sohee Lee, Youngsup Song and Sang-Wook Kang

**Note 1:** The synthesis and unique topology of N7a-PrP-AV3 was analyzed in a cell-free translation system based on rabbit reticulocyte lysate, in the presence of rough microsome derived from HeLa cells (Fig. S1A and S1B) and verified to produce ctmPrP (Fig. S2D), whose C-terminal region is glycosylated (Fig. S2A and S3B) and N-terminal region is exposed to the cytosolic side (Fig. S2C). These experiments not only provided an experimental idea for the reconstitution of the mechanism involved in the synthesis of each PrP isoform but also led us to propose the mutant-selective topologic conversion (MSTC) as a mechanism of ctmPrP generation (Fig. S1C).

**Note 2:** In this experiment, we first labeled all newly synthesized proteins in the cells expressing N7a-PrP-AV3 with [^35^S]-methionine. Cytosolic proteins were then extracted from pulse-labeled cells via selectively permeabilizing the plasma membrane using a low concentration of digitonin (0.015%). Semi-permeabilized cells with intact ER membranes were then incubated with PrP-A antibody. Because this antibody specifically recognizes the very end of the N-terminal region of PrP ^1^, we reasoned that ctmPrP featuring a cytosolically-exposed N-terminal region could be selectively recovered by this antibody (Fig. 1B).

**Note 3:** ERAD is an ATP-driven mechanism that extracts substrates from the ER membrane to the cytosol for transfer to the proteasome ^2^. This mechanism is triggered by Cdc48/p97, a well-known binding partner of Bag6 and required for the extraction of substrates from ER membrane ^3, 4^. Therefore, we reasoned that suppressing Cdc48/p97 would delay the degradation of ctmPrP, as long as it is an ERAD substrate. This was verified by the observation that suppression of Cdc48/p97 delayed degradation of ctmPrP (Fig. S3C and S3D). Conclusively, secPrP and ctmPrP are metabolized in the opposite directions, where MSTC selectively routes ctmPrP to the proteasome for degradation via ERAD. To this end, MSTC allows ctmPrP to remain in the ER transiently with degradation-competent topology.

**Note 4:** Sequential detergent extraction, followed by ultracentrifugation, yielded five fractions enriched in cytosolic proteins (“Pre-extract”), non-ribosome-associated membrane proteins (“Non-RAMPs”), ribosomes (“Ribosomes”), and soluble and insoluble ribosome-associated-membrane proteins (“Sol- & Insol-RAMPs”) ^5^ (Fig. S6). Ribosomes and RAMPs were further solubilized with TX-114 (~1%) in high salt (~500 mM NaCl) to release ribosomes from the translocon complex, and they were recovered by ultracentrifugation (“Ribosomes”). The remaining translocon complexes (“RAMPs”) were further separated into soluble (“Sol-RAMPs”) and insoluble fractions (“Insol-RAMPs”) by TX-114 phase-separation. Successful fractionation was verified by monitoring the subcellular markers’ distribution via immunoblotting (Fig. 4F). Of the five fractions analyzed, the ribosome fraction was the only one in which the PrP nascent chain was detected. Eventually, we successfully isolated the RNCs *in vivo*. In this fractionation, we found that translocon core components, Sec61α and Sec61β, and a translocon auxiliary component, TRAPα, were recovered in the fraction enriched in insoluble ribosome-associated membrane proteins (“Insol-RAMPs”). These components are well-known membrane proteins that bind directly to translating ribosomes ^6, 7^.

**Note 5:** The internal hydrophobic region of the PrP (i.e., PrP106-126) is highly conserved among the various species and is considered one of the most critical domains involved in the conformational conversion of the normal cellular form to the pathogenic scrapie form ^8, 9^. Within this region, a point mutation (A117V) disrupting the palindromic sequence (-AGAAAAGA-) in PrP is highly amyloidogenic and associated with prion disease ^10^. There were comparable results for the AV3 mutation (PrP-AV3) which were verified by detergent insolubility (Fig. S7B) and incomplete trypsin digestion (Fig. S7A).

**Note 6:** Nevertheless, viability of MSTC cells was lower than that of wtPrP cells. Among the various possible reasons for this paradoxical observation, the most logical explanation would be the proteotoxicity of cytosolic PrP (cytPrP) rerouted from the ER membrane during MSTC (Fig. 2A and Fig. S1B). In addition to ctmPrP, non-glycosylated cytPrP was synthesized transiently before degradation in our pulse-chase experiments of cells expressing N7a-PrP-AV3 (Fig. 2A) and was detected more from N7a-PrP-AV3 than from wtPrP in the *in vitro* analyses (Fig. S1B) and in the cells upon proteasome inhibition (Fig. 2A). Given the fact that both cytPrP and ctmPrP were degraded by the same path and that they share the machineries involved in substrate recognition, delivery, and degradation in the proteasome, increased ctmPrP expression is likely to overwhelm the capacity of proteasomal degradation and may inhibit cytPrP clearance. Several early studies to demonstrate the cytotoxic effects of cytPrP accumulated in the cytosol support this proposed mechanism for less viability of MSTC cells compared to wtPrP cells ^11, 12, 13^.

**Note 7:** The early molecular steps of MSTC, including targeting of the signal recognition particle-dependent ribosome-nascent polypeptide to the ER membrane, and transfer to the translocon, are consistent with those of the general translocation of secretory and membrane proteins at steady-state ^14^. When signal sequence efficiency is compromised (such as the N7a-signal), the nascent PrP polypeptides carrying pathogenic mutations within their internal hydrophobic domain (such as A117V and its equivalent AV3 mutation) undergo post-targeting rejection from the translocon. Eventually, they are synthesized as the ctmPrP form, the N-terminal region of which is exposed to the cytosolic side ^15, 16^. Since PrP bears a regulated signal sequence, and its translocation is attenuated during ER stress, MSTC appears to be a physiologically relevant process as part of the preemptive quality control (pQC) pathway ^17^. In the present study, we discovered an additional topologic sequence required for MSTC within the cytosolically-exposed N-terminal region of ctmPrP (Fig. 3). This sequence works cooperatively with metal-binding octapeptide repeats (OPR) during MSTC, and spatially interferes with the interaction of the nascent PrP polypeptide with BiP (Fig. 4) that acts as a molecular ratchet, supporting protein translocation into the ER ^18, 19^. In this context, MSTC appears to be achieved through various trans-acting factors that spatiotemporally engage the signal sequence, OPR, and polycationic cluster in a topology-dependent manner. Here we focused on the N-terminal polycationic cluster identified as a novel topologic sequence.

**Note 8:** PrP is a GPI-anchored protein and is generally eliminated by the endo-lysosomal pathway rather than ERAD when it is misfolded ^20^. In the present study, we provide several lines of evidence to suggest that ctmPrP is an ERAD substrate. First, ctmPrP is sensitive to proteasome but not lysosome activity (Fig. 2). Second, the unprocessed GPI-anchored sequence allows ctmPrP to be extracted from the ER membrane to the cytosol (Fig. S4D). Finally, after extraction, the ctmPrP is delivered to the proteasome via the p97/Bag6-dependent pathway (Fig. S3D and Fig. 2E). The N-terminal polycationic cluster is actively involved in the ctmPrP triage decision at three mechanistically distinctive steps. First, the N-terminal polycationic cluster is an important sequence requirement for the MSTC (Fig. 4). Second, its cytosolic exposure is provided both as an ER-retention signal and as an amino-terminal degradation signal, like a degron, for ctmPrP (Fig. 3). Eventually, ctmPrP is discriminated from other PrP isoforms and rapidly degraded via the proteasome-dependent pathway.

**Note 9:** The N-terminal polycationic cluster has been physiologically implicated in a number of PrP functions, including PrP internalization^21^, glucosaminoglycan binding ^22^, and interactions with tubulin ^23^ and GPCR-Adgrg6 ^24^, and has received special interest because its neutralizing antibodies ^25^ and deletion mutations ^26^ yield dramatically delayed disease and prion propagation. However, the PrP’s pathogenic effect is still questionable because most of these studies were performed using deletion mutants or recombinant proteins without considering PrP isoforms undergoing conformational conversion. Because the unique PrP topology is determined during translocation into the ER ^15, 16^, we focused on ctmPrP, which is identified as the only form closely associated with spontaneous neurodegeneration ^27, 28^. Although it remains to be examined how MSTC contributes to the pathogenic consequence of extracellular PrP, our study demonstrates that MSTC prevents expression and accumulation of several fatal PrPs with mutations in their internal hydrophobic domain (Fig. 5). In PrP-AV3’s case, MSTC captures AV3 in the ER membrane’s lipid bilayer and prevents its exposure to the lumen. Bypassing MSTC for PrP-AV3, caused by the KA3 mutation, liberates AV3 from the ER membrane and exposes it to the secretory pathway. In this context, the logical sequence of events is that PrP-AV3 liberated from the ER membrane presents in a misfolded state on the cell surface. However, misfolded PrP-AV3 is discriminated on the cell surface, internalized rapidly from the cell surface, and eventually degraded in the lysosome by “RESET” pathway ^20^. RESET provides a logical explanation for viable recovery of the cells expressing proteotoxic PrP-AV3 (Fig. S7C).

**References**

1. Emerman AB, Zhang ZR, Chakrabarti O, Hegde RS. Compartment-restricted biotinylation reveals novel features of prion protein metabolism in vivo. *Molecular biology of the cell* 2010, **21**(24)**:** 4325-4337.

2. Hegde RS, Ploegh HL. Quality and quantity control at the endoplasmic reticulum. *Current opinion in cell biology* 2010, **22**(4)**:** 437-446.

3. Ye Y, Meyer HH, Rapoport TA. The AAA ATPase Cdc48/p97 and its partners transport proteins from the ER into the cytosol. *Nature* 2001, **414**(6864)**:** 652-656.

4. Kadowaki H, Nagai A, Maruyama T, Takami Y, Satrimafitrah P, Kato H*, et al.* Pre-emptive Quality Control Protects the ER from Protein Overload via the Proximity of ERAD Components and SRP. *Cell reports* 2015, **13**(5)**:** 944-956.

5. Conti BJ, Devaraneni PK, Yang Z, David LL, Skach WR. Cotranslational stabilization of Sec62/63 within the ER Sec61 translocon is controlled by distinct substrate-driven translocation events. *Molecular cell* 2015, **58**(2)**:** 269-283.

6. Gogala M, Becker T, Beatrix B, Armache JP, Barrio-Garcia C, Berninghausen O*, et al.* Structures of the Sec61 complex engaged in nascent peptide translocation or membrane insertion. *Nature* 2014, **506**(7486)**:** 107-110.

7. Voorhees RM, Fernandez IS, Scheres SH, Hegde RS. Structure of the Mammalian Ribosome-Sec61 Complex to 3.4 A Resolution. *Cell* 2014.

8. Muramoto T, Scott M, Cohen FE, Prusiner SB. Recombinant scrapie-like prion protein of 106 amino acids is soluble. *Proc Natl Acad Sci U S A* 1996, **93**(26)**:** 15457-15462.

9. Brandner S, Isenmann S, Raeber A, Fischer M, Sailer A, Kobayashi Y*, et al.* Normal host prion protein necessary for scrapie-induced neurotoxicity. *Nature* 1996, **379**(6563)**:** 339-343.

10. Ning L, Wang Q, Zheng Y, Liu H, Yao X. Effects of the A117V mutation on the folding and aggregation of palindromic sequences (PrP113-120) in prion: insights from replica exchange molecular dynamics simulations. *Mol Biosyst* 2015, **11**(2)**:** 647-655.

11. Rane NS, Kang SW, Chakrabarti O, Feigenbaum L, Hegde RS. Reduced translocation of nascent prion protein during ER stress contributes to neurodegeneration. *Developmental cell* 2008, **15**(3)**:** 359-370.

12. Ma J, Wollmann R, Lindquist S. Neurotoxicity and neurodegeneration when PrP accumulates in the cytosol. *Science* 2002, **298**(5599)**:** 1781-1785.

13. Rambold AS, Miesbauer M, Rapaport D, Bartke T, Baier M, Winklhofer KF*, et al.* Association of Bcl-2 with misfolded prion protein is linked to the toxic potential of cytosolic PrP. *Molecular biology of the cell* 2006, **17**(8)**:** 3356-3368.

14. Kim SJ, Mitra D, Salerno JR, Hegde RS. Signal sequences control gating of the protein translocation channel in a substrate-specific manner. *Developmental cell* 2002, **2**(2)**:** 207-217.

15. Kim SJ, Rahbar R, Hegde RS. Combinatorial control of prion protein biogenesis by the signal sequence and transmembrane domain. *The Journal of biological chemistry* 2001, **276**(28)**:** 26132-26140.

16. Kim SJ, Hegde RS. Cotranslational partitioning of nascent prion protein into multiple populations at the translocation channel. *Molecular biology of the cell* 2002, **13**(11)**:** 3775-3786.

17. Kang SW, Rane NS, Kim SJ, Garrison JL, Taunton J, Hegde RS. Substrate-specific translocational attenuation during ER stress defines a pre-emptive quality control pathway. *Cell* 2006, **127**(5)**:** 999-1013.

18. Cesaratto F, Sasset L, Myers MP, Re A, Petris G, Burrone OR. BiP/GRP78 Mediates ERAD Targeting of Proteins Produced by Membrane-Bound Ribosomes Stalled at the STOP-Codon. *J Mol Biol* 2018.

19. Matlack KE, Misselwitz B, Plath K, Rapoport TA. BiP acts as a molecular ratchet during posttranslational transport of prepro-alpha factor across the ER membrane. *Cell* 1999, **97**(5)**:** 553-564.

20. Satpute-Krishnan P, Ajinkya M, Bhat S, Itakura E, Hegde RS, Lippincott-Schwartz J. ER Stress-Induced Clearance of Misfolded GPI-Anchored Proteins via the Secretory Pathway. *Cell* 2014, **158**(3)**:** 522-533.

21. Sunyach C, Jen A, Deng J, Fitzgerald KT, Frobert Y, Grassi J*, et al.* The mechanism of internalization of glycosylphosphatidylinositol-anchored prion protein. *The EMBO journal* 2003, **22**(14)**:** 3591-3601.

22. Pan T, Wong BS, Liu T, Li R, Petersen RB, Sy MS. Cell-surface prion protein interacts with glycosaminoglycans. *Biochem J* 2002, **368**(Pt 1)**:** 81-90.

23. Osiecka KM, Nieznanska H, Skowronek KJ, Karolczak J, Schneider G, Nieznanski K. Prion protein region 23-32 interacts with tubulin and inhibits microtubule assembly. *Proteins* 2009, **77**(2)**:** 279-296.

24. Kuffer A, Lakkaraju AK, Mogha A, Petersen SC, Airich K, Doucerain C*, et al.* The prion protein is an agonistic ligand of the G protein-coupled receptor Adgrg6. *Nature* 2016, **536**(7617)**:** 464-468.

25. Moroncini G, Kanu N, Solforosi L, Abalos G, Telling GC, Head M*, et al.* Motif-grafted antibodies containing the replicative interface of cellular PrP are specific for PrPSc. *Proc Natl Acad Sci U S A* 2004, **101**(28)**:** 10404-10409.

26. Turnbaugh JA, Unterberger U, Saa P, Massignan T, Fluharty BR, Bowman FP*, et al.* The N-terminal, polybasic region of PrP(C) dictates the efficiency of prion propagation by binding to PrP(Sc). *The Journal of neuroscience : the official journal of the Society for Neuroscience* 2012, **32**(26)**:** 8817-8830.

27. Hegde RS, Mastrianni JA, Scott MR, DeFea KA, Tremblay P, Torchia M*, et al.* A transmembrane form of the prion protein in neurodegenerative disease. *Science* 1998, **279**(5352)**:** 827-834.

28. Chakrabarti O, Hegde RS. Functional depletion of mahogunin by cytosolically exposed prion protein contributes to neurodegeneration. *Cell* 2009, **137**(6)**:** 1136-1147.
