# Supplementary material for: Mutant-selective topologic conversion facilitates selective degradation of a pathogenic prion isoform
Source: Cell Death Differ. 2019 May 24;27(1):284–96. doi: 10.1038/s41418-019-0354-1 (PMC7205900; doi:10.1038/s41418-019-0354-1)
Supplement: Supplementary file 2 — Supplementary Figure Legends [file 41418_2019_354_MOESM2_ESM.docx]

**[Supplemental Data]**

**Mutant-selective topologic conversion facilitates selective degradation of a pathogenic prion isoform**

Yumi Lee, Hongsik Eum, Duri Lee, Sohee Lee, Youngsup Song and Sang-Wook Kang

**Figure S1.** Various topologic isoforms of PrP were produced by the combinatorial control between signal sequence and internal hydrophobic domain, in vitro. PrPs fused with different signal sequences (A) in a combination of AV3 mutation (B) were translated in rabbit reticulocyte lysate in the presence of rough microsomes derived from HeLa cells. Their membrane topologies (C) were determined by proteinase K (PK) protection assay. Of note, differently than other PrPs, PrP containing N7a signal sequence was completely digested by PK, indicating the failure of translocation. In contrast, in a combination of AV3 mutation, a product that spans membrane was synthesized, as shown by the generation of a small fragment that resistant to PK.

**Figure S2.** The unique membrane topology of ctmPrP was determined in vitro. In vitro translation of N7a-PrP-AV3 in the presence of RM resulted in the synthesis of two PrP products (Fig. S2B). Between them, the upper band proved to be ctmPrP as shown by the results as follows. First, the upper band was selectively captured by immobilized Con A, indicating the glycosylated form of PrP (i.e., Con A is a lectin known to be associated with glycosylated proteins) (A). Second, the PK-resistant fragment was sensitive to glycosidase (i.e., PNGase F), indicating that C-terminal region bearing two glycosylation sites is in the ER lumen (B). At last, the PK-resistant fragment failed to be captured by PrP-A antibody reactive to very end of flexible N-terminal region, whereas it is successfully captured by an antibody reactive to 3F4 epitope close to the internal hydrophobic region of PrP (see also Fig S1) (C). These data together suggest that N7a-PrP-AV3 successfully synthesizes ctmPrP of which N-terminal region is exposed to the cytosolic side and C-terminal region is in the ER with spanning membrane (D).

**Figure S3.** Cells stably expressing PrP-AV3s that generate ctmPrP (N7a) and cytosolic PrP (N3) were cultured in the presence of MG132 (A) or BAF-A1 (B), fully solubilized at indicated time points, and subjected to immunoblotting with 3F4 antibody. Of note, N7a-PrP-AV3 was increased by MG132 in a rate similar to cytosolic PrP, but not by BAF-A1, indicating that it is degraded by the proteasome-dependent pathway. When p97 expression was suppressed by shRNA (shp97) (C), newly synthesized ctmPrP was somewhat increased, and its degradation was delayed (D).

**Figure S4.** The presence of unprocessed signal sequence and GPI-anchored sequence at both ends of ctmPrP was examined in vitro. Truncated mRNAs as indicated were translated in vitro with RMs and analyzed on the gel. Of note, in the size basis, nascent N7a-PrP-AV3 polypeptides migrated slightly slower than Prl-PrP, providing the possibility that one or both of signal sequences at both end of ctmPrP is unprocessed (A). To examine whether GPI-anchored sequence is unprocessed, we created various PrP constructs containing or lacking signal sequence or GPI-anchored sequence without perturbing ctmPrP topogenesis (B, upper panel). PrPs were translated with RMs in vitro and digested by PK. N-linked glycan modifications of PK-resistant C-terminal fragments were removed by PNGase F. Of note, the gel mobility of the product generated from N7a-PrP (AV3) was similar to that of SA-PrPs containing or lacking N7a-signal but slightly slower than that of ctmPrP lacking GPI-anchored signal (B, lower panel). To investigate whether N7a-PrP-AV3 is linked to membrane, we translated N7a-PrP-AV3 (N7a/AV3) and Prl-PrPs containing or lacking GPI-anchored sequence were translated in vitro with RMs. RMs were solubilized in TX-114 (1%) and separated into non-detergent (upper) and detergent phase (lower) (C). Of note, Whereas Prl-PrP linked to membrane via GPI modification was recovered in the detergent phase, N7a-PrP-AV3 was recovered in non-detergent phase where Prl-PrP lacking GPI-anchored sequence was recovered (D). Uncleaved signal sequence was determined by similar experiment as in (B), but its size was compared without any enzymatic processing (E). These data together suggest that N7a-PrP-AV3 bears unprocessed signal sequence and GPI-anchored sequence at its both ends (F).

**Figure S5.** Effect of N-terminal polycationic cluster on MSTC was examined by the modification of G34N. We created mutant N7a-PrP-AV3 constructs carrying G34N (“CHO*”) whose individual lysine residue within N-terminal polycationic cluster was replaced with alanine (A). HeLa cells expressing these constructs were pulse-labeled, and newly synthesized PrP was immunoprecipitated by 3F4 antibody (B). Lysine residues of N7a-PrP-AV3 lacking G34N were replaced in the same manner. Pulse-labelled HeLa cells expressing these constructs were subjected to immunoprecipitation at 2 hr after chase (C). Of note, compared with other mutation, the levels of newly synthesized K27A and KA3 were obviously increased, and their considerable amount still remained after chase (C).

**Figure S6.** Strategy for ribosome-associated membrane protein (RAMP) isolation. Isogenic Flp-In T-Rex 293 cells stably expressing ctmPrP and KA3 mutant were used for the isolation of RAMPs. Detergents and salt concentrations used in this study were indicated along each arrow.

**Figure S7.** Detergent (DB buffer) lysates of the cells stably expressing wtPrP and PrP-AV3 were subjected to analyses of trypsin sensitivity and detergent solubility. Trypsin sensitivity was determined by monitoring the residual PrPs that resistant to trypsin (0~0.25%) (A). Detergent solubility was determined by comparative analysis of PrP level in soluble and insoluble fractions separated by centrifugation (B). Of note, although PrP-AV3 appeared to be biochemically more proteotoxic than wtPrP as shown by reduced sensitive to trypsin and solubility in detergent, the numbers and size of colonies of viable cells were similar to the cells expressing wtPrP (C).
