# Supplementary figures and images for: Mutant-selective topologic conversion facilitates selective degradation of a pathogenic prion isoform
Source: Cell Death Differ. 2019 May 24;27(1):284–96. doi: 10.1038/s41418-019-0354-1 (PMC7205900; doi:10.1038/s41418-019-0354-1)

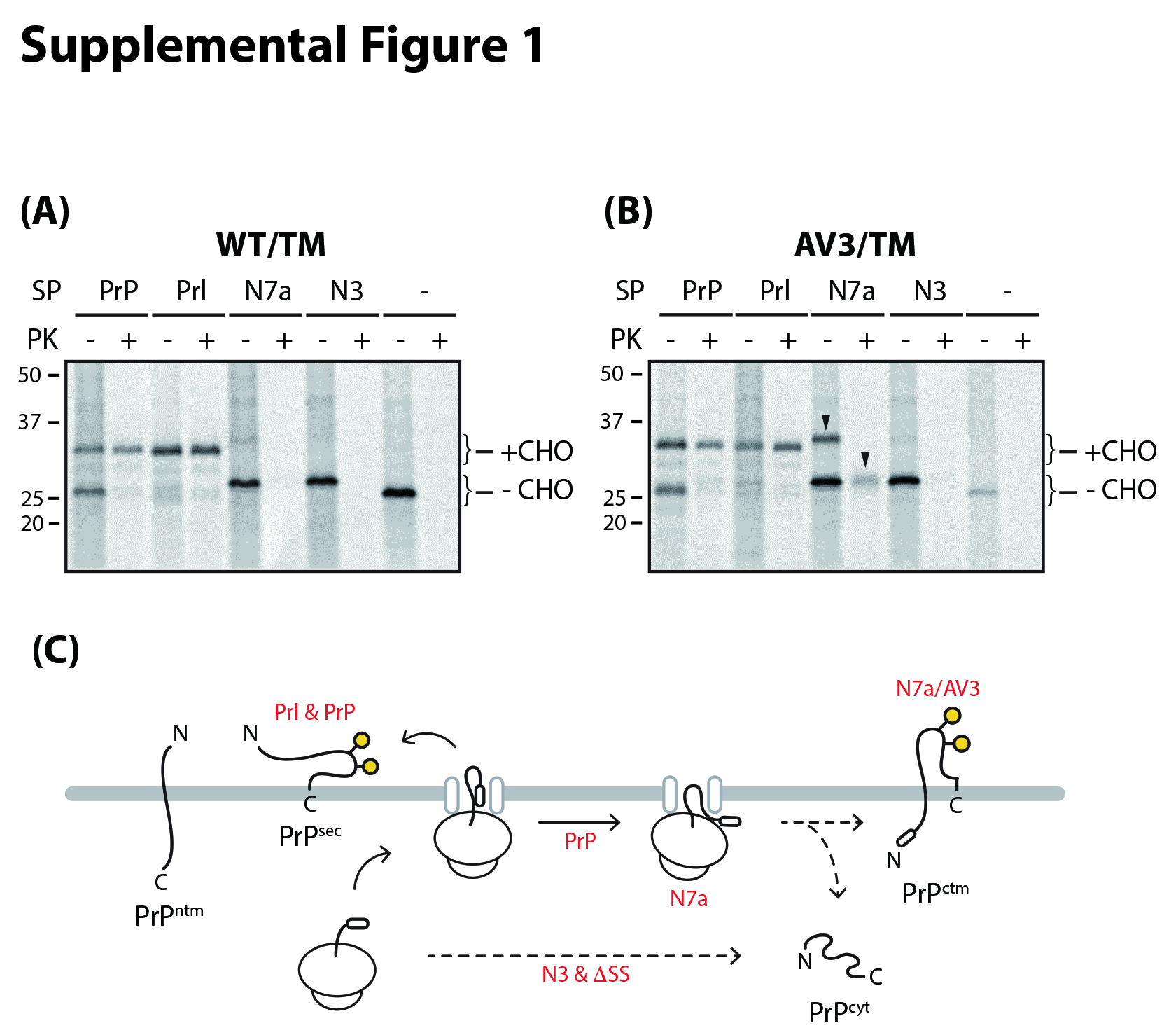

Supplement: Supplementary file 3 — Supplementary Figure 1 [file 41418_2019_354_MOESM3_ESM.jpg]

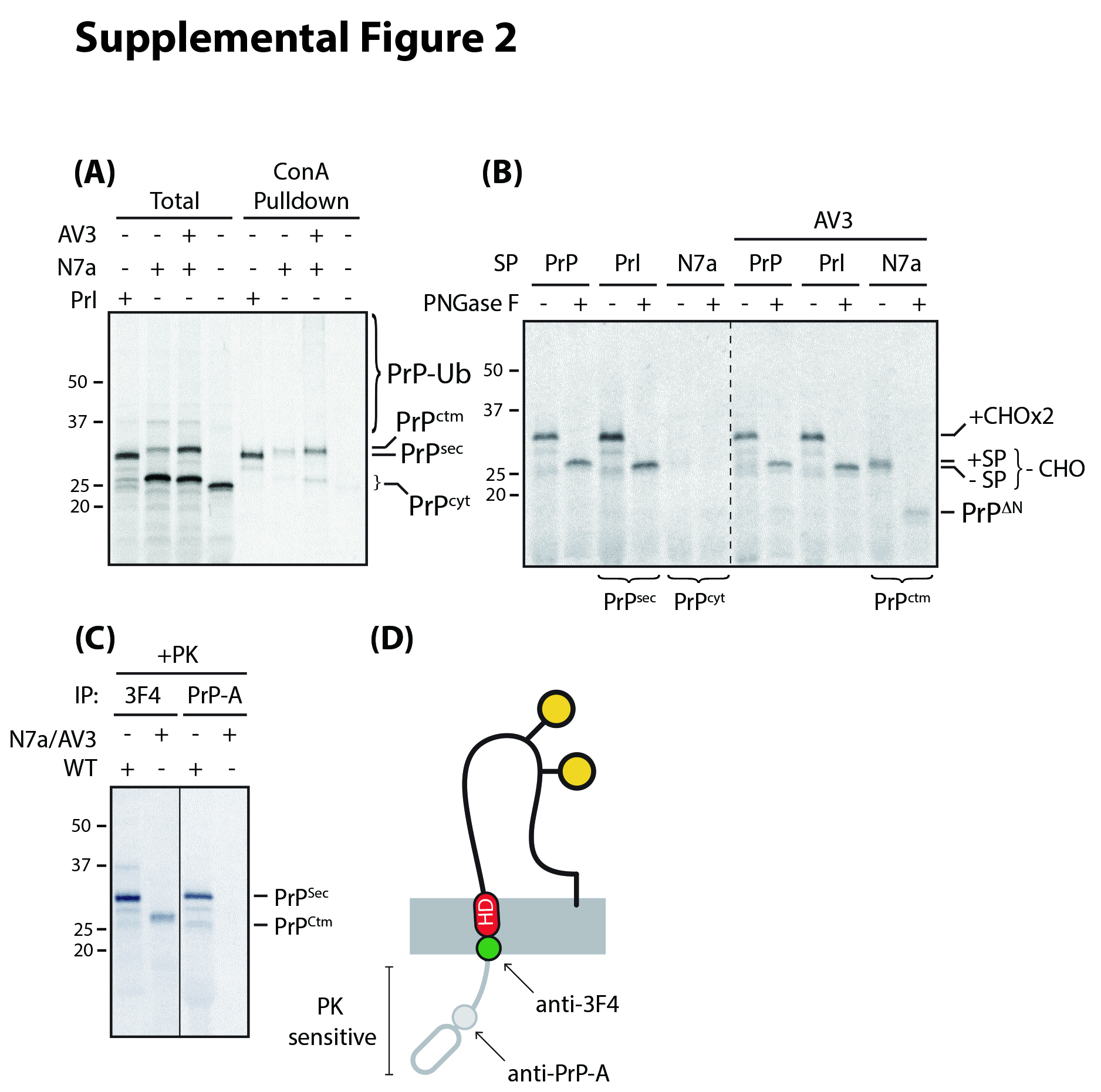

Supplement: Supplementary file 4 — Supplementary Figure 2 [file 41418_2019_354_MOESM4_ESM.jpg]

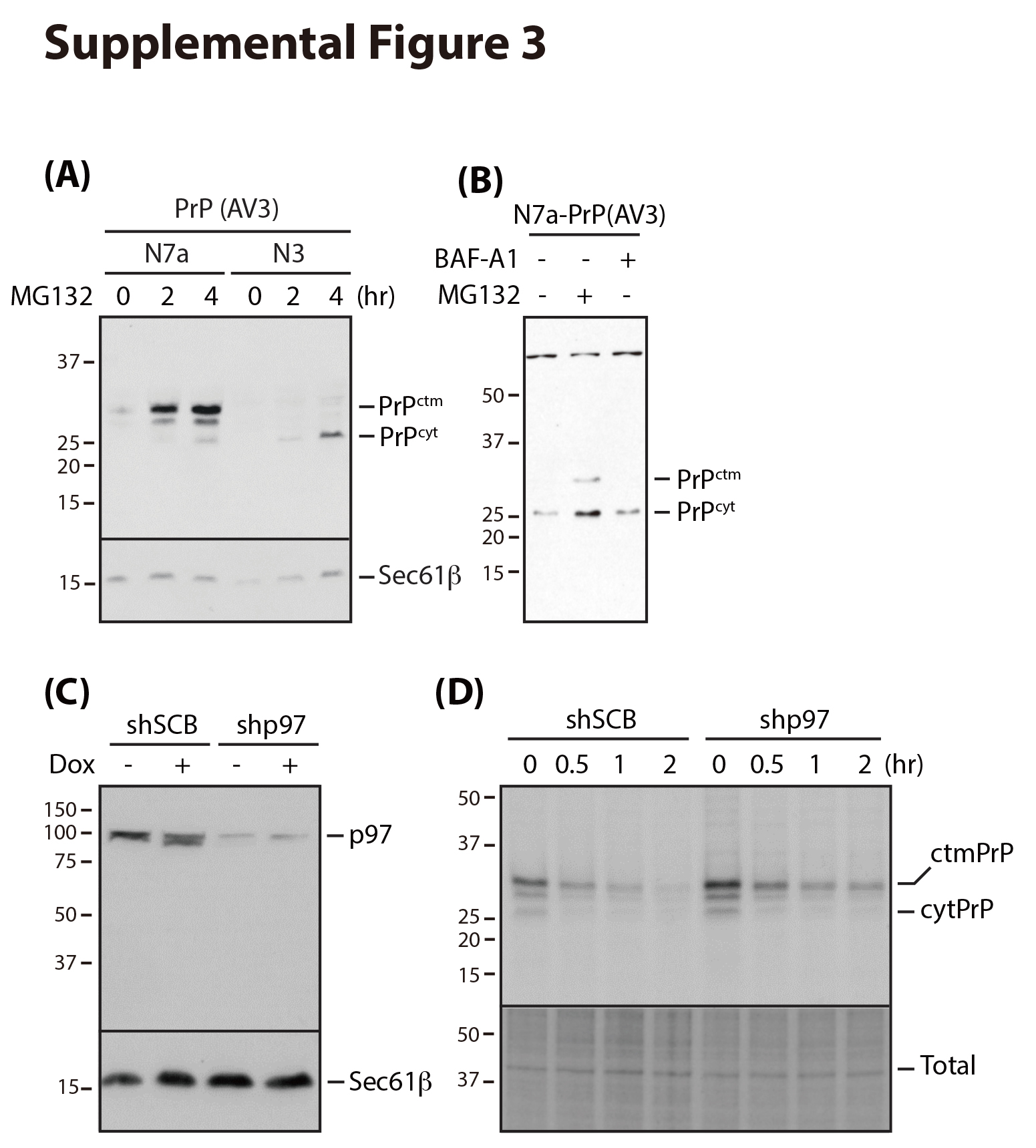

Supplement: Supplementary file 5 — Supplementary Figure 3 [file 41418_2019_354_MOESM5_ESM.jpg]

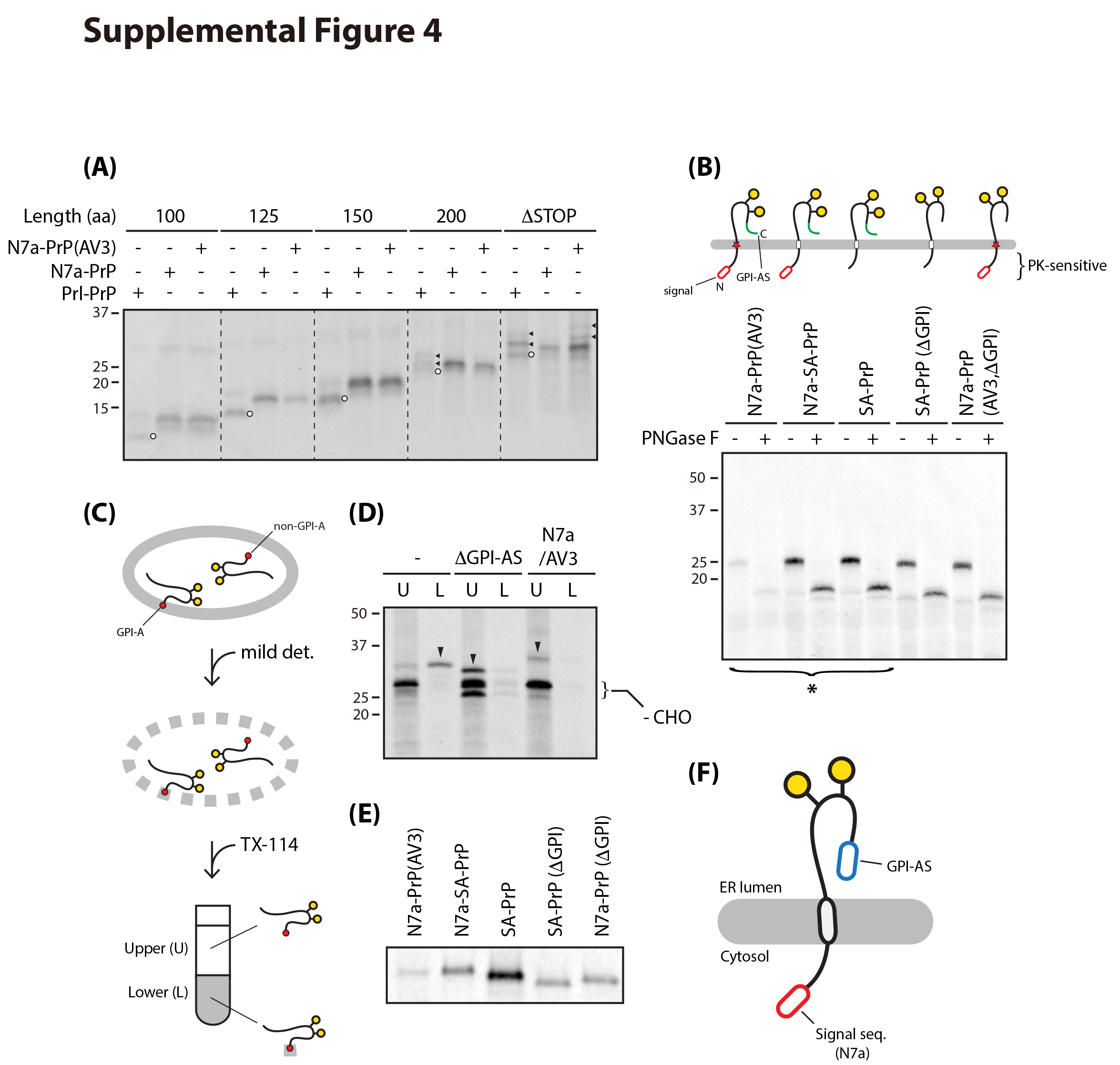

Supplement: Supplementary file 6 — Supplementary Figure 4 [file 41418_2019_354_MOESM6_ESM.jpg]

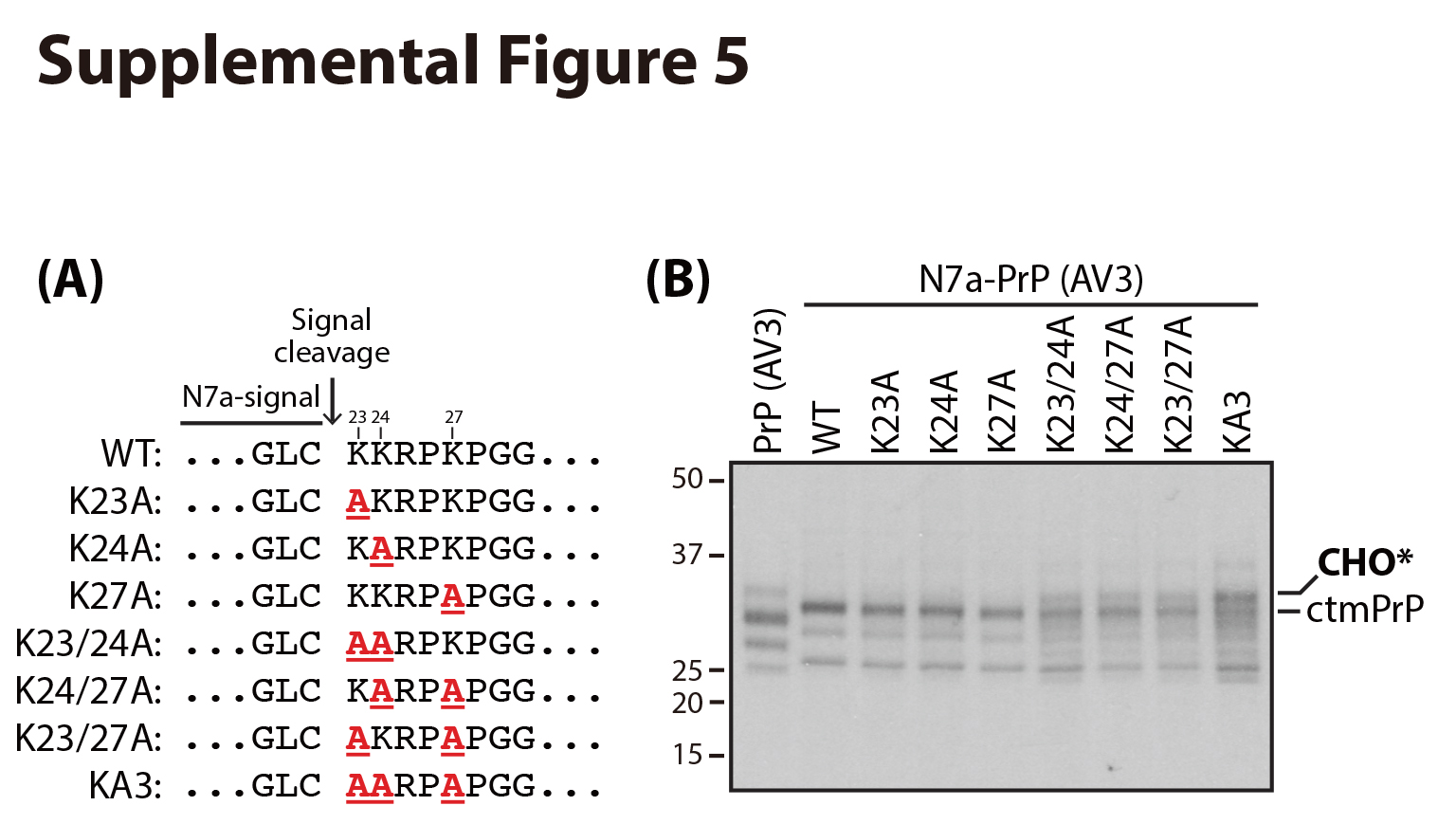

Supplement: Supplementary file 7 — Supplementary Figure 5 [file 41418_2019_354_MOESM7_ESM.jpg]

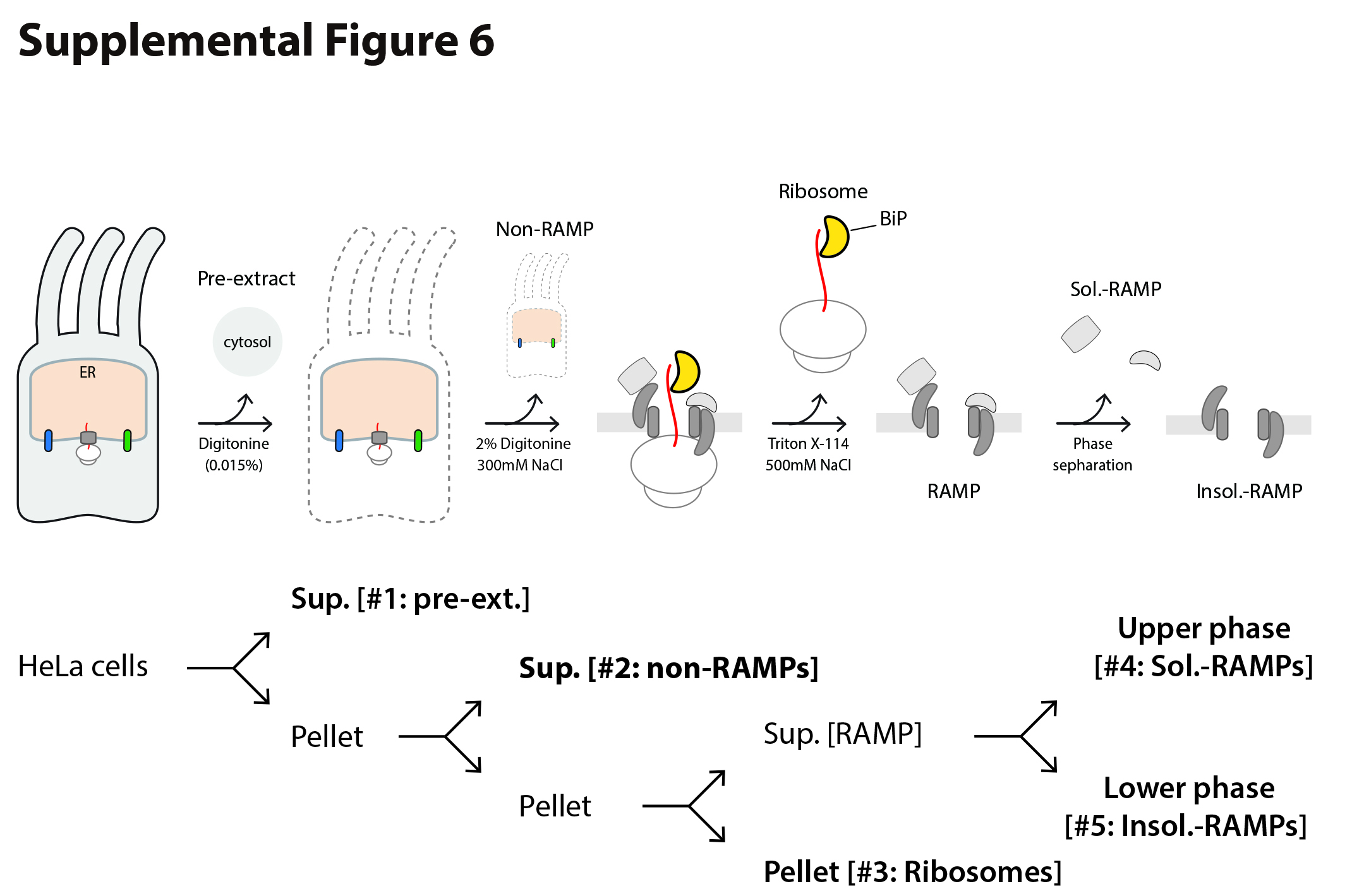

Supplement: Supplementary file 8 — Supplementary Figure 6 [file 41418_2019_354_MOESM8_ESM.jpg]

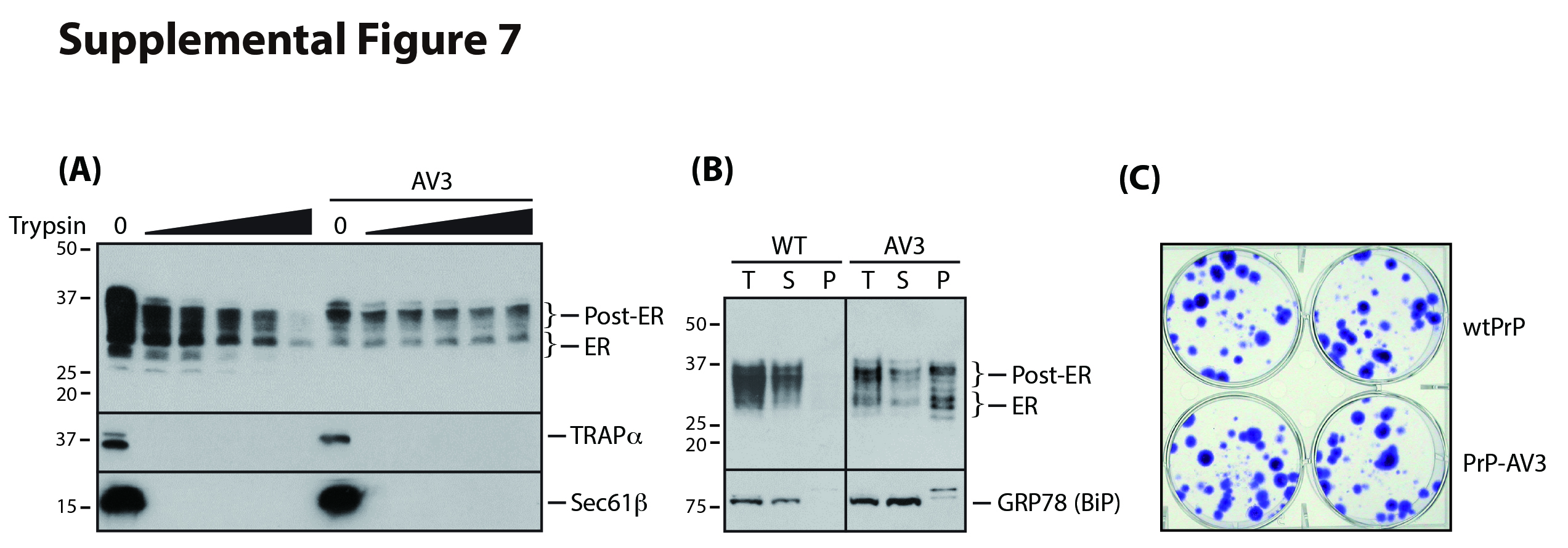

Supplement: Supplementary file 9 — Supplementary Figure 7 [file 41418_2019_354_MOESM9_ESM.jpg]
